# Universal chromatin state annotation of the mouse genome

**DOI:** 10.1186/s13059-023-02994-x

**Published:** 2023-06-27

**Authors:** Ha Vu, Jason Ernst

**Affiliations:** 1 Bioinformatics Interdepartmental Program, University of California, Los Angeles, CA 90095 USA; 2 Department of Biological Chemistry, University of California, Los Angeles, Los Angeles, CA 90095 USA; 3 Eli and Edythe Broad Center of Regenerative Medicine and Stem Cell Research at University of California, Los Angeles, Los Angeles, CA 90095 USA; 4 Computer Science Department, University of California, Los Angeles, Los Angeles, CA 90095 USA; 5 Jonsson Comprehensive Cancer Center, University of California, Los Angeles, Los Angeles, CA 90095 USA; 6 Molecular Biology Institute, University of California, Los Angeles, Los Angeles, CA 90095 USA; 7 Department of Computational Medicine, University of California, Los Angeles, Los Angeles, CA 90095 USA

## Abstract

A large-scale application of the “stacked modeling” approach for chromatin state discovery previously provides a single “universal” chromatin state annotation of the *human* genome based jointly on data from many cell and tissue types. Here, we produce an analogous chromatin state annotation for *mouse* based on 901 datasets assaying 14 chromatin marks in 26 cell or tissue types. To characterize each chromatin state, we relate the states to external annotations and compare them to analogously defined *human* states. We expect the universal chromatin state annotation for *mouse* to be a useful resource for studying this key model organism’s genome.

**Supplementary Information:**

The online version contains supplementary material available at 10.1186/s13059-023-02994-x.

## Background

Mouse is widely adopted as a model organism for human for many reasons, including its genetic and physiological proximity to humans, relatively short life span, and availability as test subjects for genetic manipulations [1–3]. A wealth of epigenomic datasets in mouse, including maps of histone modifications and variants and sites of accessible DNA, has accumulated thanks to efforts from different consortia and individual labs, which can be used to annotate the mouse genome, including non-coding regions [4–10]. This type of data has previously been integrated through methods such as ChromHMM and Segway [11–14] to generate chromatin state maps for various organisms’ different cell and tissue types [6, 15–19]. These chromatin state maps have traditionally been used to annotate genomes in a per-cell-type manner, using either the “independent” or “concatenated” modeling approaches (for ease of presentation, we will refer to tissue types also as cell types) [14, 20].

Recently, we applied an alternative “stacked” modeling approach of ChromHMM to learn chromatin states from over 1000 human datasets representing more than 100 cell types, to generate a universal annotation of the human genome that can be shared across human cell types [21]. This modeling approach provided a single annotation of the genome per position based on data from all the input cell types. Such an annotation, denoted full-stack annotation, offers complementary advantages to per-cell-type annotations, such as differentiating constitutively active regions from cell-type-specific ones and simplifying genome annotations across cell types through a single annotation shared across cell types, as opposed to one for each cell type. Additionally, the full-stack annotation allows researchers to bypass picking a single cell type for analyses or conducting analyses separately for every cell type. This can be particularly useful in studies involving data that is not inherently cell-type-specific such as analyses of genetic variants or conserved DNA sequence. However, an analogous full-stack annotation has not been previously available in mouse.

To address this, we train a full-stack model with ChromHMM using input data from > 900 mouse datasets of 14 chromatin marks from 26 mouse cell type groups (“Methods” section). We analyze these states with respect to their enrichments with external datasets and annotations to provide detailed characterizations for each state. We also analyze to what extent each mouse state shows enrichment for human states at sequence mapped positions. We expect the mouse full-stack annotations, along with the provided biological characterizations, will be a useful resource for studying this key model organism.

## Results and discussion

**Fig. 1:**
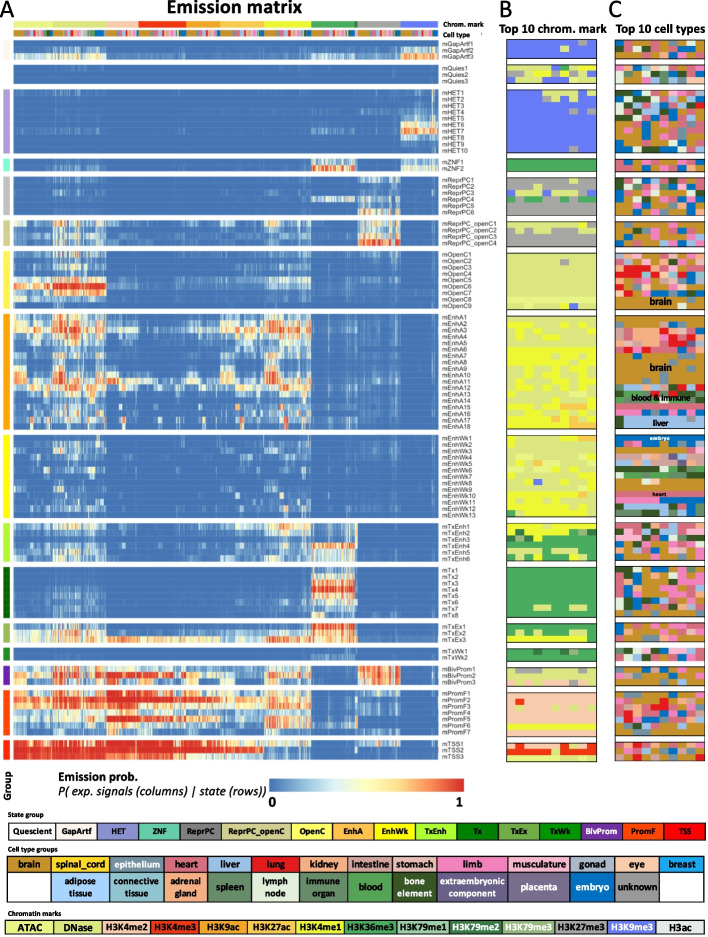
Mouse full-stack state emission parameters.** A** Each of the 100 rows in the heatmap corresponds to a mouse full-stack state. Each of the 901 columns corresponds to one input dataset. For each state and each dataset, the heatmap gives the probability within the state of observing a binary present call for the dataset’s signal. Above the heatmap, the first color bar indicates the assay/chromatin mark measured by each dataset. The second color bar shows the cell type groups associated with each dataset. The corresponding color legends are shown towards the bottom. The states are displayed in 16 groups based on biological interpretations indicated by the color legend at the bottom, with white space between each group. Full characterization of states is available in Additional file 2. The model’s transition parameters between states can be found in Additional file 1: Fig. S1. Columns are ordered such that datasets profiling the same chromatin marks are next to each other. **B** Each row corresponds to a full-stack state as ordered in **A**. The columns correspond to the top 10 datasets with the highest emission value for each state, in order of decreasing ranks, colored by their associated chromatin marks as in **A**. **C** Similar to **B**, but datasets are colored by the associated cell type groups. The cell type groups primarily associated with some of the enhancer states are noted inside the heatmap. A fully annotated version of Fig. 2**B–C** is provided in Additional file 3

We learned the mouse full-stack model by applying ChromHMM to over 900 mouse epigenomic datasets, similar to how it was previously applied in human [12, 21] (“Methods” section, Fig. 1, Fig. S1). We used a 100-state model for consistency with the previously analyzed human full-stack model.

We manually grouped these 100 states into 16 groups. One of the groups contains states associated with assembly gaps or alignment artifacts (mGapArtf), the latter of which are often marked by signals of both open chromatin mark (ATAC or DNase) and heterochromatin mark H3K9me3 (Fig. 1). Another group, Quiescent group (mQuies), consists of states associated with minimal signals of any chromatin marks. We defined a heterochromatin (mHET) group primarily associated with H3K9me3 and a Zinc finger genes (mZNF) group associated with both H3K36me3 and H3K9me3. We also defined a Polycomb repressed group (mReprPC) associated with primarily H3K27me3 and another group (mReprPC_openC) associated with both open chromatin marks (DNase- and/or ATAC-seq) and polycomb-repressed-associated mark H3K27me3. We also defined a group of states associated with just open chromatin (mOpenC), based on high DNase-seq and ATAC-seq signals relative to other chromatin marks.

We defined three groups of states associated with candidate enhancers: active enhancers (mEnhA), weak enhancers (mEnhWk), and transcribed enhancers (mTxEnh). States in the mEnhA group were associated with open chromatin, H3K27ac and H3K4me1. States in the mEnhWk group also showed association with those marks, but at lower levels compared to those in the mEnhA group. States in the mTxEnh group showed signals of open chromatin (ATAC and/or DNase), H3K4me1, H3K27ac, *and* transcription-associated marks (H3K36me3 or H3K79me2/3).

In addition to the mTxEnh group, we defined three additional transcription groups: transcription (mTx), transcription and exons (mTxEx), and weak transcription (mTxWk). States in the mTx group are associated primarily with the transcription marks H3K36me3 and/or H3K79me2/3. Meanwhile, states in transcription and exons group (mTxEx) are associated with both open chromatin and transcription marks. States in the mTxWk group are associated with low levels of transcription marks.

We also defined three promoter-associated groups: bivalent promoters (mBivProm), promoter flanking (mPromF), and transcription start sites (mTSS). States in these groups generally had relatively high levels of H3K4me2 and H3K4me3, and for some of them also H3K4me1 and/or open chromatin marks. mBivProm states were also associated with the repressive mark H3K27me3. States in the mTSS group tended to have weaker H3K4me1 levels.

Within each group, there were differences among individual states, such as the magnitude of the emission probabilities associated with specific chromatin marks, or their association with different cell type groups (Fig. 1). For example, different states in the active (mEnhA) and weak enhancer (mEnhWk) groups have enhancer-associated marks that were specific to different cell type groups such as the brain, blood, immune, liver, and embryo (Fig. 1C). Detailed descriptions of each state’s chromatin mark signals and cell-type-specific activities are provided in Additional file 2.

**Fig. 2:**
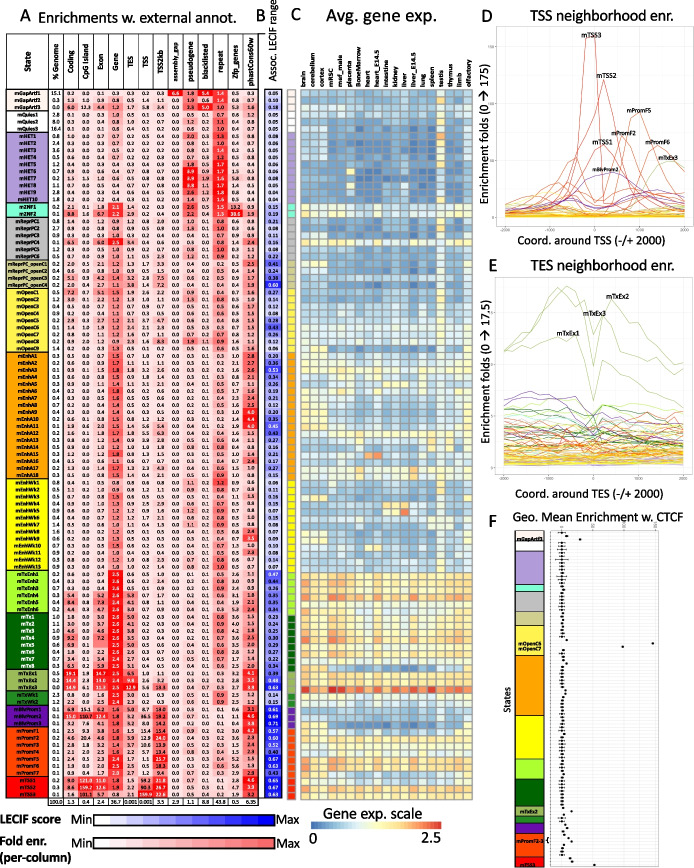
Mouse full-stack state enrichments for external genomic annotations. **A** Fold enrichments of mouse full-stack states with external genome annotations (“Methods” section). Each row corresponds to a state and each column corresponds to one external genomic annotation: coding sequences, CpG islands, exons, gene bodies (exons and introns), transcription end sites (TES), transcription start sites (TSS), TSS and 2-kb surrounding regions, assembly gaps, pseudogenes, blacklisted regions, repeat elements, annotated Zfp genes, and PhastCons conserved elements (“Methods” section). The last row shows the percentage of the genome that each external genome annotation covers. The heatmap colors are column-normalized, i.e., within each column, the colors of the cells are such that the highest values are colored red and the lowest values are colored white. **B** Each row indicates the states’ average LECIF score, indicating evidence at the functional genomics level of human-mouse conservation based on epigenetic annotations [22] (“Methods” section). The list of states with top average LECIF scores and highest enrichments with PhastCons elements is in Additional file 1: Fig. S9 and Additional file 4. **C** Average weighted expression of genes that overlap each full-stack state in different groups of cell types (“Methods” section). Each column in the heatmap corresponds to a cell group indicated at the top. Each row corresponds to a state, as ordered in **A**. **D**, **E** Positional enrichments of full-stack states relative to annotated **D** transcription start sites (TSS) and **E** transcription end sites (TES). Positive coordinate values represent the number of bases downstream in the 5′ to 3′ direction of transcription, while negative values represent the number of bases upstream. Each line shows the positional enrichments in a state. Lines are colored corresponding to the state group as indicated in **A**. **F** Geometric mean and geometric standard deviation of enrichments of full-stack states CTCF elements across 28 cell types (“Methods” section). States are displayed vertically in the same order as **A**. The mOpenC6-7 state showed the strongest enrichment for CTCF elements in all observed cell types. The geometric mean and standard deviation are calculated such that for each state, fold enrichment values of 0 are replaced by the state’s minimum non-zero value. The fold enrichment values accompanying this plot are available in Additional file 4

We also conducted various enrichment analyses to further characterize the states (Fig. 2A). Enrichments with external annotations further highlight the distinctions among states from different groups, as well as among those within the same group. For example, the state mGapArtf1 overlapped with 99.9% of annotated assembly gaps in mm10 (6.6-fold) (Fig. 2A). States mGapArtf1 and mGapArtf3 jointly overlapped with 81.1% of the blacklisted regions from ENCODE (5.4- and 5.0-fold, respectively) (Fig. 2A). States in promoter-associated groups (mTSS, mPromF, mBivProm) showed relatively high enrichments with regions within 2 kb of annotated TSSs (9.4–26.7 fold, Fig. 2A). These states vary in their enrichments with regions upstream and downstream of annotated TSSs (Fig. 2D, Additional file 1: Fig. S2). The three states from the TSS group (mTSS1-3) had the strongest enrichment for TSS (59.2–159.9 fold). These three states along with mBivProm2 were strongly enriched with CpG islands (101.1–159.2 folds, Fig. 2A). States in the transcription-associated groups (mTx, mTxWk, mTxEnh, mTxEx) all had enrichments greater than 2.4-fold for annotated gene bodies. States in the transcription and exon group (mTxEx1-3) showed the highest enrichments for annotated exons (11.3–14.7 folds, Fig. 2A) and regions surrounding annotated TESs (Fig. 2E, Additional file 1: Fig. S2). States mOpenC6-7, which had strong *constitutive* DNase-seq and/or ATAC-seq signal while having relatively limited histone modification signals, had the strongest enrichments with CTCF binding sites in multiple cell types (geometric mean 146- and 98-fold for states mOpenC6-7, respectively) (Figs. 1 and 2F, Additional file 3).

Additionally, we analyzed the enrichment of full-stack states for different chromosomes. This uncovered three states in the polycomb repressed group (mReprPC4-6) that were highly enriched on chromosome X (8.9–11.4 fold, Additional file 1: Fig. S3), likely related to H3K27me3-associated chromosome X inactivation [23, 24]. We also found chromosome Y strongly enriched for mGapArtf1 state (6.4 fold, corresponding to 96% of chrY) (Additional file 1: Fig. S3).

We also analyzed the states’ enrichments for different classes of repeat elements [25]. For the two largest classes of repeats, long interspersed nuclear elements (LINE) and long tandem repeats (LTRs) (Additional file 1: Fig. S4-5), the most enriched states were both in the HET group (mHET9 and mHet7) (2.7- and 3.3-fold, respectively). Satellite and rRNA repeats had the strongest enrichments for the mGapArtf3 state, 22.5- and 95.5-fold, respectively.

We also related the full-stack states to the average expression of overlapping genes (“Methods” section). States in the transcription-associated groups (mTxEnh, mTx, mTxEx), along with those related to the promoter (mPromF and mTSS groups) tended to be associated with higher average gene expression across cell types compared to other groups (Fig. 2C). State mTxEx3 was associated with the highest gene expression of all states.

Additionally, we analyzed the mouse full-stack states’ association with per-cell-type chromatin state annotations defined across 66 reference epigenomes from 12 unique cell type groups and 7 developmental stages, based on 8 marks (Additional file 1: Fig. S6-7, Additional file 5) [19]. This revealed, for example, that state mEnhA17 showed the strongest enrichments with per-cell-type active enhancer states across all developmental stages for the liver (Additional file 1: Fig. S6-7, Additional file 5), which is consistent with this state’s highest signals in enhancer-associated chromatin marks (H3K4me1, H3K27ac) for liver datasets (Fig. 1, Additional file 3). State mTSS2 was most enriched with per-cell-type active promoter states in all reference epigenomes (Additional file 1: Fig. S6-7, Additional file 5), consistent with its association with individual chromatin marks (Fig. 1, Additional file 3).

In addition, we analyzed how the mouse full-stack states correspond to those of an analogous previously defined full-stack model in human [21]. We evaluated the enrichments of each mouse full-stack state with each human full-stack state after mapping the human annotations to mouse based on DNA sequence information (“Methods” section). Sixty-four and 22 states showed > ten-fold and > 50-fold enrichment, respectively, with at least one human state (Additional file 1: Fig. S8-9, Additional file 4) [21], and these states’ biological implications highlight strong correspondence of states from the human and mouse models. For example, mouse state mTxEx3 showed 378.8-fold enrichment for human state TxEx4—the largest enrichment across any pair of states—(Additional file 1: Fig. S9, Additional file 4). These two states showed the highest average gene expression across multiple mouse and human cell types, respectively [21] (Fig. 2C). All 13 mouse states in the promoter groups (mPromF, mBivProm, mTSS states) showed strong enrichments with human full-stack states that are also promoter-associated, with 12 of these mouse states showing > 90-fold enrichment (Additional file 1: Fig. S9, Additional file 4). Mouse states mOpenC6-7 showed the strongest association with the human DNase state, and all these states are associated with *constitutive* open chromatin and CTCF elements (Fig. 2F, Additional file 3) [21]. However, there exist differences between states from the two organisms’ models. For example, the state group mReprPC_OpenC (showing DNase-seq and/or ATAC-seq and repressive mark H3K27me3 signals) was only observed in the mouse model, while the group Acet (showing signal primarily for a diverse set of acetylation marks) was only present in the human model, which was as expected as most of these acetylation marks were not in the mouse model input. Seven states in the mOpenC group (all except mOpenC6-7) showed *cell-type-specific* signals of open chromatin, a pattern not observed in the human model (Additional file 1: Fig. S8-9, Additional file 4). Other states such as those in the mouse mHET and human HET groups shared an association with H3K9me3 and enrichments for repetitive elements, but did not show strong enrichments in this analysis. This was expected because the mapping of human and mouse states was based on corresponding DNA sequences between the two species.

We also evaluated each full-stack state’s average human-mouse LECIF score, which quantifies evidence of conservation at the functional genomics level between a pair of sequence-aligned positions from the two species and is bounded between 0 and 1 (Fig. 2B) [22] (“Methods” section). Here, the average LECIF score in each state ranged from 0.04 (mHET9) to 0.71 (mBivProm3) (Fig. 2B, Additional file 1: Fig. S9). All 14 mouse states that had an average LECIF score ≥ 0.5 also had a > 50-fold enrichment with a human full-stack state, highlighting that mouse states with high LECIF scores show concordance with specific human states. In addition, we looked at each state’s enrichment for sequence constraint elements as defined by PhastCons [26]. Across all states, the states’ enrichments for PhastCons elements and average LECIF score showed overall consistency (Spearman correlation 0.70; *p*-value: 3.8e^ −16 ^). We found 10 mouse states that are among the top 20 states based on (1) average LECIF score, (2) enrichments for PhastCons element, and (3) enrichments for a specific human full-stack state (Additional file 1: Fig. S9). Among these states, seven are associated with promoter activities (mBivProm1-3, mTSS1-3, mPromF1), two states are characterized by strong exon enrichments and constitutive transcriptional activities (mTxEx2-3), and one state (mEnhA3) corresponds to constitutively strong candidate enhancers (Additional file 1: Fig. S9). Interestingly, a few states stand out as associated with either high sequence constraint or functional conservation (LECIF score), but not in both. For example, constitutive DNase-candidate insulator states mOpenC6-7 are among the top 20 with the highest average LECIF scores yet had lower (PhastCons) sequence constraint enrichment (ranked 50, 59) (Additional file 1: Fig. S9).

## Conclusions

We introduced the mouse full-stack annotation to provide a universal chromatin state map using > 900 epigenomic datasets from 26 cell type groups. The mouse full-stack model and its characterization are analogous to the previous human full-stack model [21] (Availability of data and materials, Additional file 2). As discussed previously, the full-stack model has a number of advantages, such as being able to differentiate constitutive from cell type-specific annotations and simplifying the overall genome annotation across cell types, in that there is a single genome annotation per position [21]. However, this does come at a trade-off of a more complex set of model parameters and potential loss of modeling power when one is interested in a specific cell type. The full-stack annotation is not meant to replace existing per-cell-type annotations, but rather to complement them and the most appropriate annotation will likely depend on the application [21]. Here, we applied the stacked modeling approach to data from different cells types, though the same modeling approach could be applied to data measured within the same cell type but under different treatments to potentially uncover latent enhancers [27]. We expect the full-stack model to serve as an additional resource for work that leverages mouse as a model organism to gain insight into human biology.

## Methods

### Input data and processing

We obtained mouse data generated by the ENCODE or mouse ENCODE projects from the ENCODE Project Portal [5, 6, 28] restricted to those files with “File analysis title” starting with “ENCODE4” and “File assembly” of “mm10.” In total, we downloaded data of read alignment (.bam files) for 901 experiments, 114 of which were DNase-seq, 83 were ATAC-seq, and 704 were ChIP-seq data targeting 12 chromatin marks representing 26 cell type groups (Additional file 2). For each .bam file resulting from a ChIP-seq assay, we extracted the corresponding control .bam file by matching the .fastq files of reads from the ChIP-seq assay with the control reads. As the DNase-seq or ATAC-seq experiments did not have paired control .bam files, we assumed a uniform background read distribution. Links to download all input data for the stacked model are provided in Additional file 2.

We then constructed the cell_mark_file input table required by ChromHMM BinarizeBam such that there are four tab-delimited columns in the table. The first column is set as “Genome” across all rows. The second column denotes the experiment names of the form “ < Biosample term name > _ < Experiment target > _ < Experiment accession > ,” where “Biosample term name,” “Experiment target,” and “Experiment accession” correspond to the cell type, the histone mark/DNase/ATAC profiled, and the accession code of such experiments, respectively, from the metadata from ENCODE. The third column contains the experiments’ .bam file names. The last column contains the matched control .bam file names, which is left blank for DNase-seq or ATAC-seq experiments, since we assumed a uniform background distribution for these assays.

Using this cell_mark_file input table, we next binarized the data at 200 base pair resolution using the BinarizeBam and MergeBinary commands of ChromHMM (v.1.23), following the procedures of [21]. 

### Training the full-stack model and generating genome-wide state annotations

We learned the mouse full-stack chromatin state model for the 901 datasets using the LearnModel command of ChromHMM (v.1.23). We applied the same set of flags as in learning the human full-stack model (-splitrows -holdcolumnorder -pseudo -many -p 6 -n 300 -d -1 -lowmem -gzip), described in Vu and Ernst [21]. We trained models with numbers of states ranging from 5 to 120, in increments of 5 states. We then calculated the negative log-likelihood, Akaike information criterion (AIC), and Bayesian information criterion (BIC) measures on a random set of 300 genomic regions, each of length 1Mbp, for all models (Additional file 1: Fig. S10). These measures show how well each model fit with the observed data, while also putting penalty on the model complexity (more states, more model parameters and hence more complex models). We observed that as the number of states increase, the negative log-likehood, AIC, and BIC measures (the lower the better) all decreased, but at diminishing magnitude (Additional file 1: Fig. S10). We decided to focus on analyzing a model with 100 states since it is similar to the number of states in the human model, to facilitate comparisons between the two models [21]. Additionally, using the ChromHMM CompareModels function, we also calculated the maximum correlation between each state in the 100-state model presented here, with a state from each of the alternative models (with different numbers of states) (Additional file 1: Fig. S10). The correlation was calculated across emission probabilities for the 901 experiments. The reported correlations indicate how well each state in the chosen 100-state model can be captured by one state in each of the alternative models. We observed that the minimum correlations between the 100-state model with any other alternative models range from 0.20 (5-state model) to 0.85 (120-state model). 

### Enrichment and estimated probabilities of overlap with per-cell-type chromatin state annotations

We obtained per-cell-type 15-chromatin state annotations for 66 reference epigenomes/cell types from Gorkin et al. [19], with download links provided in Kwon and Ernst [22]. For simplicity, we use the terms “reference epigenome” and “cell types” interchangeably, and we refer to the chromatin state segmentation that is used to annotate the individual reference epigenomes as per-cell-type annotations. This model was trained using the *concatenated* modeling approach from data of 8 chromatin marks measured in 12 cell type groups at up to 8 distinct stages during mouse fetal development [19]. We applied the same procedure as outlined in Vu and Ernst [21] to obtain two types of summary results of the relationship between mouse full-stack states with states in per-cell-type annotations. First, for each full-stack state, we report, for each of the 64 reference epigenomes, the chromatin state from the per-cell-type model that is maximally enriched in the full-stack state [21]. Second, for each of the 12 tissue types, we report the estimated probabilities of each full-stack state overlapping with each of the 15 states in the per-cell-type model [21]. These results, along with detailed comments about the observed patterns of overlap between each full-stack state and per-cell-type state, are available in Additional file 5. Data of all per-cell-type annotations were in mm10 [19].

### Average gene expression associated with each full-stack state

We obtained data of gene expression for 19 tissue types in mouse from Ref. [29] (http://chromosome.sdsc.edu/mouse/download/19-tissues-expr.zip). The provided data contains two gene expression datasets for each tissue type, corresponding to two replicates. We converted the gene expression values for the 19 tissues into ${log}_{2} (FPKM+1)$ values, where FPKM (fragments per kilo base of transcript per million mapped fragments) were the provided values from the source data, and we added a pseudo count of 1 for each value.

Since the gene expression data was provided in mm9, we lifted the mouse full-stack annotation from mm10 to mm9. To do so, we first wrote the full-stack annotation in mm10 into a .bed file such that each line corresponds to a 200-bp segment. We then used the liftOver tool with default parameters to convert the 200-bp segments from mm10 to mm9. We filtered out regions in the lifted-over mm9 annotation that were mapped from ≥ 2 distinct segments in mm10.

For each full-stack state and each of the gene expression datasets (there are 38 of them with 2 replicates for each tissue type), we calculated the average gene expression of all genes that overlap with the state, while taking into account the genes’ length. We followed the same procedure described in Vu and Ernst [21]. In particular, within a dataset, let the length and expression of gene $g$ be denoted ${L}_{g}$ and ${E}_{g}$, respectively. Let ${B}_{s}$ be the set of 200-bp genomic segments $i$’s that are assigned to state $s$ in the mouse full-stack annotation, in mm9. Let ${G}_{i}$ denote the set of genes that overlap with genomic segment $i$. The gene-length-normalized average expression for state $s$ is calculated as done previously [21]:

$${avg\,exp\,bp\,normalized}_s=\frac{\sum_{i\in B_s}\sum_{g\in G_i}\frac{E_g}{L_g}}{\sum_{i\in B_s}\sum_{g\in G_i}\frac1{L_g}}$$

We then obtained the average gene expression for each full-stack state in each dataset. To calculate the average gene expression for the states in each of the 19 tissue types, we averaged the calculated average expression across the two replicate datasets for the same tissue type.

### Overlap enrichments between mouse and human full-stack states

We first lifted over the human full-stack annotation from hg19 to mm10, using the procedure previously described in Vu and Ernst [21], such that each genomic bin of size 200-bp was independently mapped from hg19 to mm10, and any genomic segments in hg19 that were mapped to the same location in mm10 were excluded from the analysis. Then, we used the ChromHMM OverlapEnrichment function to calculate the overlap fold enrichment between each human state (liftedOver to mm10) and each mouse state. 

For each pair of human-mouse states, we determined whether the pair of states correspond to a one-to-one mapping by checking whether the human state was most enriched with the mouse state compared to the other 99 human states and vice versa for the mouse state.

### External annotation sources

The sources for external annotations for enrichment analyses are as follows (all download links are listed in Additional file 2).

- Annotations of CpG islands, exons, gene bodies (exons and introns), transcription start sites (TSS), and transcription end sites (TES), 2-kb windows surrounding TSSs (TSS2kb) in mm10 were RefSeq annotations included in ChromHMM (v 1.23) and originally based on annotations obtained from the UCSC genome browser [30, 31] on July 26, 2015.
- Annotation of coding gene regions corresponds to coordinates of genes whose feature type is “CDS” from GENCODE mm10 gene annotation, vM25 [32], accessed on February 3, 2022.
- Annotation of assembly gaps in mm10 was obtained from the UCSC genome browser and corresponds to the Gap track [30, 31], accessed on February 3, 2022.
- Annotations of pseudogenes in mm10 correspond to coordinates of genes whose gene type or transcript type contained “pseudogene” from GENCODE’s mm10 gene annotation, vM25 [32].
- Blacklisted regions were downloaded from ENCODE project portal in mm10 from [33]. We note that two states stood out in their enrichments with blacklisted regions, GapArtf1 and GapArt3. In total, 413,502,000 bp were annotated as either state GapArtf1 or GapArtf3, and 238,977,200 bp are annotated as the blacklisted region. The Jaccard index between the blacklisted region and GapArtf1/3 regions is 0.42 (193,765,600 bp of intersection, constituting 81.1% of all blacklisted regions, and 458,713,600 bp of union).
- Annotations of different repeat classes were downloaded from the UCSC genome browser repeat masker track in mm10, accessed on Jan. 14, 2022 [25].
- Annotations of Zinc finger genes in the mouse genome correspond to the coordinates of genes whose name contained “Zfp” based on GENCODE mm10 annotation vM25 [32].
- Annotations of different chromosomes’ coordinates were downloaded from the UCSC genome browser’s data of chromosome sizes in mm10, from https://hgdownload.gi.ucsc.edu/goldenPath/mm10/bigZips/mm10.chrom.sizes [30, 31].
- LECIF scores measure the human-mouse evidence of conservation at the functional genomics level and were downloaded in version 1.1 from https://github.com/ernstlab/LECIF [22]. For each full-stack state, we reported the average LECIF score of overlapping genomic bases with the state.
- CTCF peaks data for mouse were downloaded as .bed files format from the ENCODE portal [5, 6, 34–36]. We only included data files that have “File analysis title” starting with ENCODE4 based on the metadata. In total, we obtained data of CTCF peaks for 42 ChIP-seq experiments from profiling CTCF in 28 unique biosamples. Details and download links for CTCF peaks data are available in Additional file 2.
- PhastCons conserved elements [26] based on the 60-way multi-species sequence alignment were downloaded from the UCSC genome browser (https://hgdownload.soe.ucsc.edu/goldenPath/mm10/database/phastConsElements60way.txt.gz).

## Supplementary Information

**Additional file 1: Supplementary Figure 1.** Mouse full-stack states transition probabilities. **Supplementary Figure 2.** Positional enrichments of full-stack states around annotated transcription start sites and transcription end sites. **Supplementary Figure 3.** Mouse full-stack states enrichments with different chromosomes. **Supplementary Figure 4.** Mouse full-stack states enrichments with different classes of repeats. **Supplementary Figure 5.** Enrichment of select mouse full-stack states with different classes of repeat elements. **Supplementary Figure 6.** Full-stack states maximum-enrichments with annotated concatenated-model chromatin states in 66 mouse reference epigenomes. **Supplementary Figure 7.** Estimated probabilities of per-cell-type concatenated-model chromatin states overlapping with mouse full-stack states. **Supplementary Figure 8.** Enrichments of mouse full-stack states with human full-stack states. **Supplementary Figure 9.** Mouse full-stack states’ relationship with LECIF scores, human full-stack states and phastCons elements. **Supplementary Figure 10.** Analysis for the number of states.

**Additional file 2.** Metadata and download links for input data used for model learning, CTCF elements.

**Additional file 3.** Summary characterizations of mouse full-stack states.

**Additional file 4.** Mouse full-stack states’ average LECIF scores, enrichments with human full-stack states, repeat classes, chromosomes and CTCF elements.

**Additional file 5.** Mouse full-stack states’ relationships with per-cell-type annotations.

**Additional file 6.** Review history.

## Data Availability

The mouse full-stack chromatin state annotation, along with detailed instructions for viewing the annotation on the UCSC Genome Browser through the provided trackhub link, is available at https://github.com/ernstlab/mouse_fullStack_annotations in mm10. The code to analyze the full-stack states is available at https://github.com/ernstlab/mouse_fullStack_annotations [37], under the MIT Open Access License. An archival version of this code and the full-stack annotation is available at https://zenodo.org/record/8011434 [38] under the MIT Open Access License. No custom scripts and software were used other than those mentioned in the “Methods” section. All download links for input for the mouse full-stack model are available in Additional file 2.
