# Supplementary material for: Universal chromatin state annotation of the mouse genome
Source: Genome Biol. 2023 Jun 27;24:153. doi: 10.1186/s13059-023-02994-x (PMC10294404; doi:10.1186/s13059-023-02994-x)
Supplement: Supplementary file 1 — Additional file 1: Supplementary Figure 1. Mouse full-stack states transition probabilities. Supplementary Figure 2. Positional enrichments of full-stack states around annotated transcription start sites and transcription end sites. Supplementary Figure 3. Mouse full-stack states enrichments with different chromosomes. Supplementary Figure 4. Mouse full-stack states enrichments with different classes of repeats. Supplementary Figure 5. Enrichment of select mouse full-stack states with different classes of repeat elements. Supplementary Figure 6. Full-stack states maximum-enrichments with annotated concatenated-model chromatin states in 66 mouse reference epigenomes. Supplementary Figure 7. Estimated probabilities of per-cell-type concatenated-model chromatin states overlapping with mouse full-stack states. Supplementary Figure 8. Enrichments of mouse full-stack states with human full-stack states. Supplementary Figure 9. Mouse full-stack states’ relationship with LECIF scores, human full-stack states and phastCons elements. Supplementary Figure 10. Analysis for the number of states. [file 13059_2023_2994_MOESM1_ESM.pdf]

List of supplementary figures

Supplementary Figure 1: Mouse full-stack states transition probabilities.
Supplementary Figure 2: Positional enrichments of full-stack states around annotated transcription start sites and transcription end sites.
Supplementary Figure 3: Mouse full-stack states enrichments with different chromosomes. *(An excel sheet supporting this figure is in Additional File 4).*
Supplementary Figure 4: Mouse full-stack states enrichments with different classes of repeats. *(An excel sheet supporting this figure is in Additional File 4).*
Supplementary Figure 5: Enrichment of select mouse full-stack states with different classes of repeat elements.
Supplementary Figure 6: Full-stack states maximum-enrichments with annotated concatenated-model chromatin states in 66 mouse reference epigenomes. *(An excel sheet supporting this figure is in Additional File 5).*
Supplementary Figure 7: Estimated probabilities of per-cell-type concatenated-model chromatin states overlapping with mouse full-stack states. *(An excel sheet supporting this figure is in Additional File 5).*
Supplementary Figure 8: Enrichments of mouse full-stack states with human full-stack states. *(An excel sheet supporting this figure is in Additional File 4).*
Supplementary Figure 9: Mouse full-stack states' relationship with LECIF scores, human full-stack states and phastCons elements. *(An excel sheet supporting this figure is in Additional File 4).*
Supplementary Figure 10: Analysis for the number of states.
Note: We included references to previous publications in the legends to some supplementary figures. The full list of these references is included at the end of this document, and all of these references are included in the reference list in the main paper.

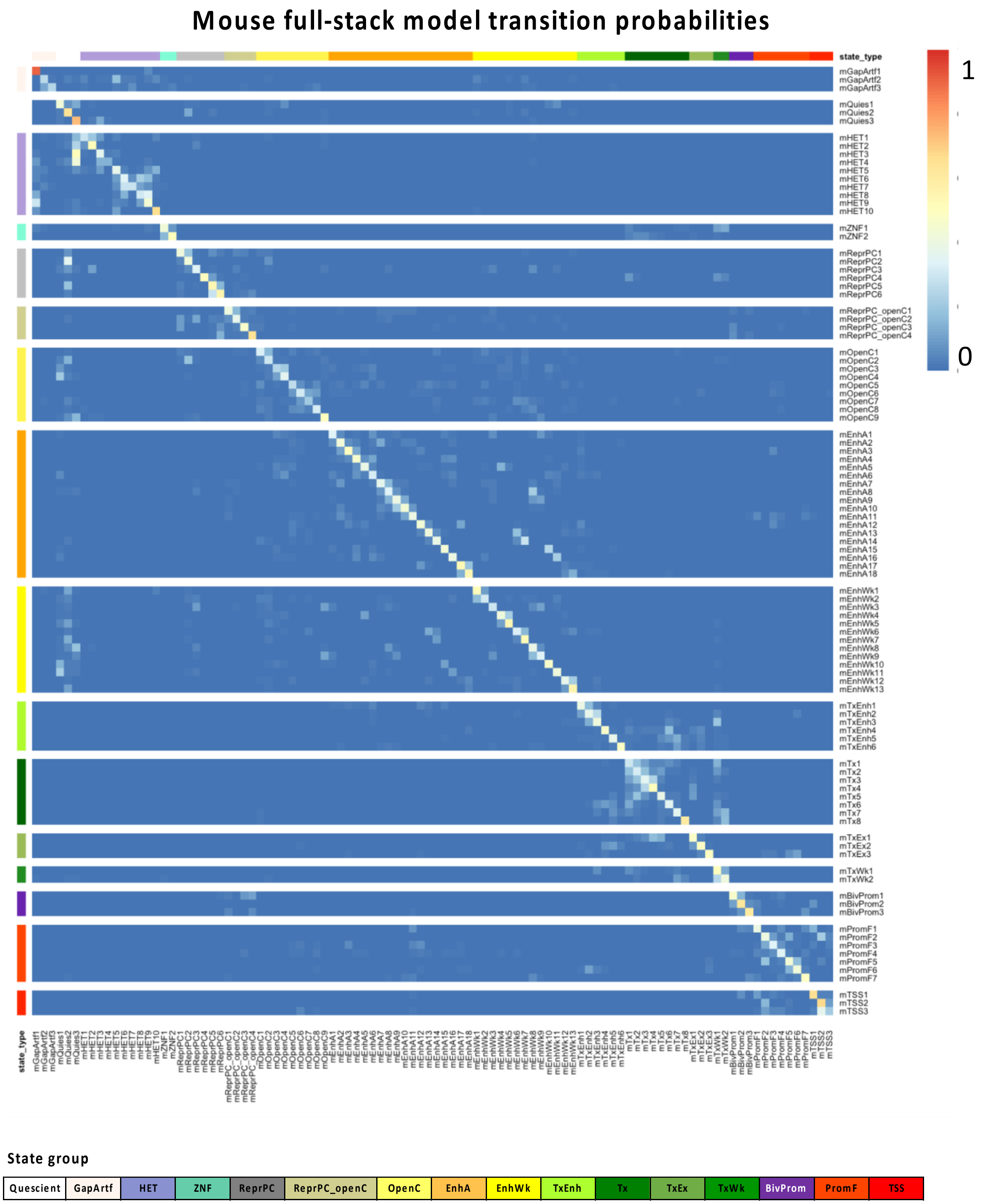

Supplementary Figure 1: Mouse full-stack states transition probabilities. Each row and each column correspond to a full-stack state, ordered based on their associated state group. The heatmap shows for each state assigned at a current genomic position (rows), the probabilities of transitioning to another state (columns) at the subsequent genomic position. The state groups are shown at the bottom.

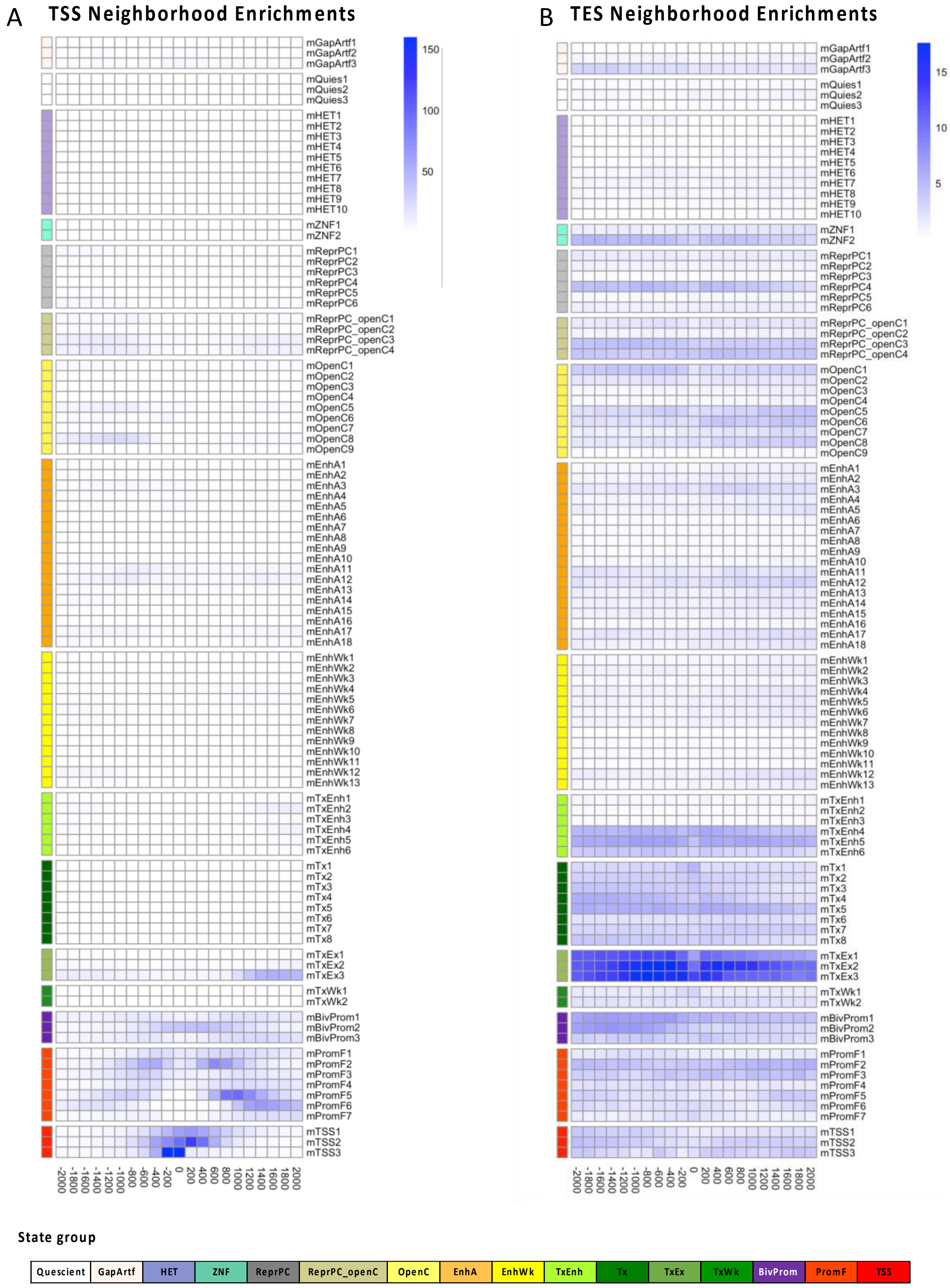

Supplementary Figure 2: Positional enrichments of full-stack states around annotated transcription start sites and transcription end sites. The figure shows positional fold enrichments for positions within 2kb of annotated (**a**) transcription start sites (TSS) and (**b**)

transcription end sites (TES). Each column corresponds to one 200bp window as indicated at bottom. Positive coordinate values represent the number of bases downstream in the 5' to 3' direction of transcription, while negative values represent the number of bases upstream. Enrichments are calculated based on a genome-wide background. Color scale of enrichments is indicated at right for each panel. State groups' color legends are shown at the bottom.

A

Enrichment of mouse full-stack states with chromosomes

state	Genome %	chr1	chr2	chr3	chr4	chr5	chr6	chr7	chr8	chr9	chr10	chr11	chr12	chr13	chr14	chr15	chr16	chr17	chr18	chr19	chrM	chrX	chrY
mGapArtf1	15.10	0.63	0.63	0.74	0.80	0.72	0.71	1.01	0.65	0.60	0.66	0.47	0.73	0.76	0.97	0.63	0.65	0.71	0.69	0.75	1.88	2.16	6.38
mGapArtf2	0.25	0.89	1.01	0.87	1.17	1.56	1.01	1.12	1.16	1.11	1.25	1.25	1.04	1.17	0.75	0.97	0.89	1.59	0.86	1.08	0.00	0.28	0.02
mGapArtf3	0.04	1.17	0.44	0.44	2.17	1.23	0.57	0.84	0.80	0.79	0.73	1.38	0.88	1.02	0.51	0.44	0.69	2.01	1.48	0.51	29.46	0.56	0.30
mQuies1	2.79	1.14	1.15	1.40	0.96	0.94	1.05	0.62	1.03	1.06	1.38	0.84	1.10	1.35	1.07	1.24	1.20	0.81	1.43	1.12	0.00	0.22	0.00
mQuies2	8.01	1.04	1.04	0.99	0.78	0.82	1.10	0.99	0.87	1.15	0.99	1.25	0.94	1.26	1.08	1.03	0.97	0.89	1.12	1.32	0.31	1.28	0.01
mQuies3	16.39	1.28	1.00	1.40	1.09	1.03	1.14	0.58	1.17	0.78	1.23	0.65	1.18	0.89	1.26	1.23	1.37	0.86	1.21	0.67	0.38	0.59	0.00
mHET1	0.81	1.12	1.10	1.12	1.14	1.57	0.96	0.82	1.46	0.95	1.41	0.62	1.08	1.06	1.13	1.20	1.11	1.30	0.98	0.49	0.00	0.06	0.00
mHET2	2.41	1.08	1.20	0.90	0.95	1.34	0.88	1.32	1.69	1.22	1.15	0.84	1.15	1.06	1.08	1.17	0.94	1.17	0.91	0.58	0.00	0.07	0.00
mHET3	3.56	1.48	0.93	1.32	1.08	1.03	1.15	0.63	1.30	0.62	1.09	0.63	1.05	0.64	1.32	1.34	1.44	1.01	1.02	0.56	2.77	0.78	0.00
mHET4	0.52	1.08	0.92	1.31	0.96	1.10	1.02	0.66	1.11	0.78	1.11	0.61	1.02	0.88	1.29	1.13	1.41	0.86	1.02	0.70	75.36	1.47	0.00
mHET5	1.23	1.27	0.88	1.17	0.93	1.20	1.03	0.96	1.07	0.93	1.12	0.71	0.89	0.96	0.92	1.09	0.99	1.19	0.90	0.61	4.01	1.39	0.06
mHET6	0.74	0.57	0.55	0.76	1.33	0.98	0.92	3.50	0.65	0.95	0.60	0.51	1.84	1.25	0.57	0.48	0.55	1.91	0.80	1.11	0.00	0.94	0.03
mHET7	0.20	0.62	0.55	0.75	1.60	1.34	0.73	2.43	0.63	0.85	0.87	0.54	1.99	1.55	0.44	0.51	0.58	1.93	1.34	0.75	0.00	0.38	0.01
mHET8	1.10	0.46	0.50	0.72	1.23	0.74	0.98	3.43	0.64	0.86	0.51	0.44	1.72	1.18	0.61	0.35	0.46	1.74	0.62	1.22	0.00	1.87	0.32
mHET9	2.80	0.66	0.58	0.88	0.91	0.65	1.03	2.02	0.66	0.77	0.61	0.45	1.11	0.93	0.77	0.49	0.56	1.29	0.64	0.91	0.00	3.35	0.85
mHET10	1.77	1.36	0.84	1.35	1.08	0.96	1.14	0.68	1.03	1.11	1.23	0.82	0.83	1.24	1.13	1.03	1.03	0.96	1.14	0.63	0.00	0.75	0.18
mZNF1	0.24	0.81	1.02	0.84	0.89	1.16	0.85	1.24	1.05	1.14	1.35	0.88	1.56	1.78	0.66	0.76	1.02	1.67	1.21	0.88	0.00	0.42	0.01
mZNF2	0.13	0.59	0.89	0.53	1.00	1.44	0.71	1.73	1.43	1.23	1.36	1.00	1.08	1.71	0.52	0.63	1.20	2.39	1.08	0.87	0.00	0.19	0.01
mReprPC1	0.76	0.69	1.39	0.51	1.45	1.28	1.01	1.38	1.14	1.42	0.58	1.99	0.72	0.85	0.69	1.08	0.79	1.32	0.59	1.69	0.00	0.56	0.00
mReprPC2	2.65	0.95	1.26	0.76	0.93	1.00	1.18	1.35	0.94	1.39	0.82	1.74	0.88	1.15	1.02	1.13	0.94	1.07	1.00	1.34	0.00	0.32	0.00
mReprPC3	0.94	0.96	1.08	0.69	1.43	1.42	0.93	1.35	1.47	1.61	0.66	1.36	1.13	1.15	1.04	0.97	0.62	0.99	0.99	0.86	0.00	0.09	0.00
mReprPC4	0.10	0.27	0.39	0.10	0.34	0.46	0.27	0.56	0.30	0.22	0.25	0.43	0.12	0.19	0.11	0.09	0.24	0.19	0.23	0.60	0.00	11.90	0.00
mReprPC5	1.21	0.43	0.56	0.37	0.48	0.54	0.37	0.48	0.56	0.58	0.33	0.47	0.43	0.71	0.53	0.22	0.33	0.33	0.38	0.91	0.00	9.22	0.00
mReprPC6	0.49	0.36	0.55	0.29	0.59	0.69	0.37	0.58	0.51	0.54	0.33	0.60	0.48	0.57	0.33	0.37	0.37	0.56	0.38	1.12	0.00	8.94	0.00
mReprPC_openC1	0.15	0.67	1.44	0.43	2.26	1.29	0.67	1.20	1.25	1.35	0.48	2.19	0.69	0.61	0.54	1.37	0.57	0.98	0.68	1.91	0.00	0.52	0.00
mReprPC_openC2	0.38	0.63	1.35	0.44	1.87	1.49	0.82	1.15	1.44	1.53	0.49	1.78	0.85	0.92	0.75	0.91	0.67	0.82	0.69	1.54	0.00	0.72	0.00
mReprPC_openC3	0.22	0.54	1.12	0.52	1.59	0.95	0.75	1.35	1.30	1.16	0.90	2.30	0.63	0.63	0.62	1.26	0.73	1.44	0.67	1.66	0.00	1.13	0.00
mReprPC_openC4	0.22	0.87	1.56	0.58	1.41	1.62	0.87	1.34	0.88	1.07	0.68	1.74	0.91	1.10	0.54	1.25	0.50	1.06	0.55	2.43	0.00	0.35	0.00
mOpenC1	0.46	0.72	1.44	0.44	1.66	1.26	0.86	1.35	1.34	1.17	0.86	2.24	0.89	0.51	0.56	1.39	0.76	1.73	0.66	1.46	0.00	0.02	0.00
mOpenC2	1.15	0.94	1.32	0.65	1.11	1.23	1.05	1.36	1.05	1.39	0.94	1.76	0.94	0.88	0.89	1.22	0.96	1.40	0.91	1.19	0.00	0.02	0.00
mOpenC3	0.43	1.06	1.11	0.90	0.92	1.06	1.10	1.06	1.05	1.26	1.17	1.26	1.04	1.27	0.98	1.10	1.04	1.29	1.30	1.27	0.00	0.12	0.00
mOpenC4	0.90	1.15	1.19	1.20	0.86	0.99	1.07	0.84	0.97	1.07	1.32	1.06	1.01	1.37	0.99	1.18	1.14	1.02	1.31	1.18	0.00	0.22	0.00
mOpenC5	0.24	0.84	1.35	0.71	1.31	1.13	0.95	1.26	1.33	1.12	1.12	1.95	1.08	0.67	0.58	1.21	0.90	1.58	0.74	1.35	0.00	0.08	0.00
mOpenC6	0.14	0.97	1.23	0.85	1.15	1.07	1.07	1.33	1.09	1.26	1.14	1.57	0.89	0.91	0.78	1.14	0.86	1.33	0.92	1.38	0.00	0.20	0.01
mOpenC7	0.20	0.97	1.13	0.84	1.06	1.27	1.10	1.13	1.01	1.30	1.05	1.63	0.88	0.89	0.91	1.08	0.92	1.22	0.92	1.26	0.00	0.46	0.01
mOpenC8	0.22	1.13	1.04	1.26	1.00	0.87	1.04	1.35	1.00	0.98	1.35	0.81	0.96	1.29	0.93	0.81	0.91	1.90	0.96	0.74	0.00	0.33	0.01
mOpenC9	1.38	1.17	1.16	1.13	0.91	1.04	1.14	0.84	1.10	1.27	1.24	0.93	1.10	1.08	1.14	1.24	1.34	0.88	1.20	0.83	0.00	0.19	0.00
mEnhA1	0.26	1.05	1.15	0.95	1.14	1.12	1.14	1.02	1.04	1.30	1.17	1.10	1.13	1.17	0.93	1.01	1.04	1.19	1.33	1.07	0.00	0.08	0.00
mEnhA2	0.24	0.97	1.41	0.77	1.38	1.18	0.99	1.00	1.29	1.27	0.89	1.75	1.20	0.81	0.77	0.98	0.79	1.29	1.18	0.95	0.00	0.06	0.00
mEnhA3	0.15	0.77	1.37	0.61	1.42	1.13	0.77	1.16	1.50	1.12	1.11	2.22	0.98	0.63	0.79	1.16	0.72	1.32	1.13	1.40	0.00	0.03	0.00
mEnhA4	0.28	0.94	1.23	0.66	1.15	1.02	1.09	1.20	1.30	1.34	1.06	1.61	0.99	1.05	0.81	1.06	1.10	1.26	1.19	1.22	0.00	0.06	0.00
mEnhA5	0.28	0.90	1.24	0.78	1.20	1.15	1.06	1.29	1.20	1.17	1.08	1.67	0.92	0.94	0.76	1.17	0.95	1.44	0.86	1.54	0.00	0.04	0.00
mEnhA6	0.40	1.10	1.14	1.01	0.99	0.95	1.28	0.99	1.15	1.20	1.22	1.08	0.99	1.22	0.95	1.09	1.08	1.01	1.41	1.19	0.00	0.12	0.00
mEnhA7	0.38	1.08	1.28	0.81	1.27	1.15	0.97	0.98	1.24	1.41	0.86	1.65	1.15	1.04	0.88	0.90	0.77	1.20	1.31	1.00	0.00	0.05	0.00
mEnhA8	0.67	1.12	1.20	1.10	1.09	1.06	1.06	0.95	1.16	1.28	0.97	1.23	1.19	1.20	1.07	0.87	1.03	0.91	1.32	1.01	0.00	0.12	0.00
mEnhA9	0.36	1.11	1.18	1.17	1.20	1.10	1.08	0.96	1.04	1.32	0.98	0.99	1.41	1.19	1.02	0.94	1.02	0.80	1.27	0.85	0.00	0.14	0.00
mEnhA10	0.20	1.07	1.29	1.03	1.29	1.10	1.02	0.95	1.08	1.38	0.88	1.26	1.42	1.20	0.91	0.89	0.88	0.86	1.29	1.00	0.00	0.10	0.00
mEnhA11	0.13	0.98	1.19	0.85	1.29	1.18	0.81	1.08	1.30	1.17	0.99	1.73	1.03	1.02	0.79	1.13	0.91	1.15	1.24	1.14	0.00	0.15	0.00
mEnhA12	0.18	0.76	1.16	0.78	1.23	1.32	0.85	1.29	1.31	1.02	1.16	2.13	0.83	0.83	0.66	1.46	0.76	1.42	0.69	1.78	0.00	0.08	0.00
mEnhA13	0.28	1.03	1.16	0.77	0.94	1.16	1.12	1.17	0.90	1.21	1.20	1.73	0.99	1.04	0.82	1.26	1.07	1.31	0.78	1.63	0.00	0.12	0.00
mEnhA14	0.46	1.11	1.12	0.83	0.90	0.99	1.09	1.11	0.81	1.34	1.18	1.84	1.06	1.13	0.86	1.24	1.12	1.41	0.73	1.35	0.00	0.12	0.00
mEnhA15	0.34	1.12	1.17	0.95	0.87	1.07	1.14	1.07	1.32	1.16	1.03	1.30	0.77	1.09	1.03	0.94	1.07	1.12	1.43	1.55	0.00	0.15	0.00
mEnhA16	0.40	1.04	1.21	0.85	1.17	1.00	1.21	1.10	1.20	1.32	1.01	1.40	0.99	1.33	1.00	0.84	0.91	1.18	1.04	1.11	0.00	0.09	0.00
mEnhA17	0.23	0.83	1.14	0.90	1.15	1.22	1.16	1.27	1.06	1.11	1.00	1.92	0.87	1.07	0.63	1.18	0.93	1.28	0.92	1.56	0.00	0.16	0.00
mEnhA18	0.31	0.99	1.13	1.00	1.07	1.03	1.19	1.10	0.77	1.12	1.03	1.55	0.95	1.41	0.69	0.92	0.99	1.09	1.15	1.55	0.00	0.47	0.00
mEnhWk1	0.80	1.16	0.93	0.95	0.95	1.12	1.14	0.97	1.22	1.16	1.29	1.34	0.84	1.14	0.95	1.05	1.16	1.19	1.09	1.07	0.00	0.33	0.00
mEnhWk2	0.45	1.03	1.16	0.82	1.16	1.27	1.07	0.96	1.19	1.27	1.23	1.64	0.94	1.05	0.86	1.01	0.93	1.35	0.93	1.20	0.00	0.06	0.00
mEnhWk3	0.47	0.91	1.11	0.71	1.43	1.41	1.06	1.22	1.05	1.71	0.81	1.44	1.15	0.95	0.90	1.25	0.84	1.11	1.03	0.84	0.00	0.04	0.00
mEnhWk4	0.41	1.02	1.17	1.02	0.96	1.15	1.14	1.11	1.04	1.25	1.12	1.42	0.80	1.08	0.87	1.08	1.04	1.16	1.12	1.59	0.00	0.15	0.00
mEnhWk5	0.87	1.14	1.02	1.19	0.91	1.13	1.18	0.95	1.13	1.13	1.17	0.99	0.88	1.32	0.97	1.14	1.11	0.92	1.16	1.67	0.00	0.20	0.00
mEnhWk6	0.42	1.25	1.15	1.07	0.85	1.04	1.19	0.93	0.73	1.08	1.36	1.40	0.96	1.24	0.84	1.11	1.23	1.06	0.75	1.49	0.00	0.31	0.01
mEnhWk7	1.37	1.34	1.06	1.11	0.80	0.84	1.15	0.90	0.79	1.14	1.36	1.24	1.00	1.35	1.02	1.11	1.27	1.27	0.89	1.26	5.41	0.22	0.01
mEnhWk8	1.57	1.14	1.08	1.34	0.99	1.01	1.16	0.77	1.13	1.04	1.22	0.87	1.22	1.11	1.20	0.94	1.36	0.88	1.30	0.93	0.00	0.24	0.00
mEnhWk9	0.62	1.16	1.09	1.25	1.04	1.12	1.16	0.80	1.11	1.20	1.22	0.77	1.22	1.11	1.09	1.14	1.30	0.88	1.23	0.83	0.00	0.16	0.00
mEnhWk10	0.73	1.13	1.09	1.12	0.89	0.98	1.29	0.82	1.12	1.21	1.12	1.03	0.95	1.43	0.99	0.99	0.97	1.09	1.42	1.36	0.00	0.23	0.00
mEnhWk11	0.92	1.16	1.15	1.29	0.98	0.96	1.13	0.86	1.11	1.16	1.17	1.03	1.07	1.37	1.15	0.98	0.94	0.91	1.36	1.10	0.00	0.11	0.00
mEnhWk12	0.35	0.93	1.15	0.88	1.05	1.23	1.12	1.17	0.93	1.19	1.04	1.81	0.95	1.05	0.74	1.07	0.91	1.35	0.91	1.68	0.00	0.20	0.00
mEnhWk13	0.90	1.15	1.09	1.13	0.90	1.03	0.99	0.81	0.68	1.10	1.01	1.38	1.12	1.29	1.01	1.15	0.93	1.17	1.00	1.58	0.00	0.64	0.01
mTxEnh1	0.24	0.73	1.30	0.63	1.27	1.39	0.86	1.18	1.27	1.18	0.98	2.10	0.96	0.76	0.87	1.48	0.82	1.44	0.74	1.42	0.00	0.01	0.00
mTxEnh2	0.36	0.89	1.21	0.84	1.22	1.28	0.95	0.98	1.10	1.26	1.01	1.86	0.96	0.95	0.79	1.35	1.01	1.29	0.97	1.21	0.00	0.11	0.00
mTxEnh3	0.75	0.96	1.27	0.95	0.98	1.07	1.05	0.83	0.92	1.30	1.01	1.66	1.06	1.03	0.87	1.36	1.09	1.10	1.11	1.42	0.00	0.28	0.00
mTxEnh4	0.25	0.77	1.31	0.67	1.27	1.35	0.79	1.34	1.04	1.20	0.96	2.28	0.85	0.79	0.78	1.16	0.99	1.38	0.86	1.53	0.00	0.08	0.00
mTxEnh5	0.37	0.78	1.28	0.55	1.17	1.41	0.85	1.31	1.50	1.27	0.99	2.06	1.03	0.60	0.64	1.22	0.89	1.59	0.80	1.41	0.00	0.04	0.00
mTxEnh6	0.19	0.88	1.36	0.72	1.51	1.34	0.89	1.16	1.28	0.94	0.88	1.85	1.01	0.69	0.77	1.26	0.90	1.31	1.23	0.87	0.00	0.10	0.00
mTx1	0.96	1.03	1.24	0.90	1.09	1.14	0.93	0.81	0.90	1.38	0.94	1.49	1.11	1.05	0.93	0.86	1.07	0.93	1.10	1.23	0.00	0.71	0.09
mTx2	1.07	1.04	1.23	0.92	1.16	1.16	0.91	0.97	0.97	1.51	0.95	1.43	1.16	1.01	0.91	0.80	1.12	1.00	1.11	1.11	0.00	0.43	0.04
mTx3	0.96	1.05	1.36	0.84	1.17	1.28	0.87	1.00	0.95	1.55	0.86	1.74	1.02	0.96	0.88	0.84	1.14	0.98	1.08	1.18	0.00	0.24	0.01
mTx4	0.91	0.98	1.36	0.74	1.23	1.48	0.82	1.16	1.11	1.41	0.89	1.91	0.93	0.93	0.71	0.93	1.16	1.00	1.00	1.29	0.00	0.04	0.00
mTx5	0.62	0.86	1.23	0.68	1.23	1.53	0.84	1.32	1.38	1.21	0.93	1.62	1.11	0.74	0.77	1.12	1.07	1.35	0.87	1.21	0.00	0.09	0.00
mTx6	0.44	0.94	1.25	0.83	1.09	1.25	1.00	0.95	1.01	1.40	0.94	1.99	1.00	0.93	0.81	0.95	1.18	1.20	1.03	1.46	0.00	0.15	0.00
mTx7	0.68	0.96	1.23	0.77	1.04	1.24	1.20	1.09	1.26	1.19	1.13	1.50	1.04	0.93	0.88	1.03	1.03	1.33	1.02	1.32	0.00	0.06	0.00
mTx8	0.35	0.94	1.29	0.83	1.21	1.27	1.30	1.34	1.15	1.63	1.03	1.09	1.35	0.75	0.55	1.17	0.92	0.99	1.05	0.74	0.00	0.12	0.00
mTxEx1	0.46	0.54	1.30	0.49	1.39	1.81	0.62	1.62	1.42	1.04	1.08	2.24	0.68	0.50	0.55	1.55	0.79	1.86	0.56	1.36	0.00	0.02	0.00
mTxEx2	0.18	0.53	1.11	0.47	1.21	1.45	0.63	1.71	1.33	1.13	1.52	2.48	0.77	0.40	0.63	1.45	0.72	2.04	0.49	1.64	0.00	0.02	0.00
mTxEx3	0.04	0.57	1.11	0.64	0.96	1.11	0.60	1.88	0.78	1.35	1.50	2.51	0.57	0.64	0.55	1.16	0.48	2.21	0.50	2.53	0.00	0.24	0.02
mTxWk1	2.32	1.11	1.22	1.08	0.95	1.00	1.04	0.76	0.85	1.23	1.10	1.09	1.08	1.29	0.98	0.98	1.06	0.96	1.11	1.32	0.00	0.69	0.06
mTxWk2	1.23	1.10	1.20	1.03	1.00	1.07	1.23	0.89	0.89	1.32	1.16	1.01	1.00	1.27	0.94	0.92	1.04	1.02	1.16	1.19	0.00	0.47	0.01
mBivProm1	0.13	0.58	1.44	0.51	1.32	1.16	1.09	1.22	1.21	0.93	0.91	2.60	0.88	0.99	0.52	1.67	0.53	1.13	0.63	1.93	0.00	0.26	0.00
mBivProm2	0.14	0.80	1.43	0.66	1.26	1.17	0.87	1.51	1.11	1.16	0.90	1.91	0.87	0.94	0.72	1.25	0.58	1.29	0.82	1.64	0.00	0.32	0.00
mBivProm3	0.09	0.99	1.50	0.90	1.13	1.34	0.81	1.69	0.98	1.29	0.80	1.35	0.79	1.12	1.10	0.91	0.49	0.78	0.76	1.10	0.00	0.58	0.00
mPromF1	0.11	0.92	1.07	1.15	1.01	1.04	0.94	1.17	1.14	1.07	1.08	1.28	1.00	0.98	1.06	0.97	0.87	0.90	1.05	0.92	0.00	1.00	0.01
mPromF2	0.19	0.70	1.17	0.77	1.11	1.15	0.82	1.56	1.25	1.22	1.10	2.20	0.73	0.90	0.67	1.14	0.81	1.63	0.68	1.85	0.00	0.15	0.00
mPromF3	0.14	0.73	1.22	0.80	1.24	1.08	0.91	1.47	1.27	1.23	1.34	2.07	0.76	0.72	0.59	1.24	0.75	1.41	0.82	1.64	0.00	0.12	0.00
mPromF4	0.14	0.91	1.07	1.02	0.96	1.04	1.14	0.96	1.10	1.11	1.34	1.33	0.89	1.26	0.76	0.96	1.05	1.07	0.98	1.36	0.00	0.73	0.02
mPromF5	0.14	0.82	1.25	0.79	1.15	1.26	0.85	1.16	1.06	1.23	0.91	1.95	0.83	1.05	0.75	1.01	0.94	1.38	0.80	1.56	0.00	0.49	0.01
mPromF6	0.13	0.86	1.27	0.88	1.16	1.22	0.87	1.16	1.12	1.25	0.92	1.96	0.83	0.95	0.72	1.01	0.94	1.42	0.85	1.45	0.00	0.35	0.00
mPromF7	0.14	1.05	1.05	1.09	1.00	0.90	1.07	1.06	1.07	1.06	1.13	0.95	0.92	1.07	1.00	1.02	0.98	0.90	1.00	1.01	0.00	1.24	0.03
mTSS1	0.15	0.81	1.17	0.92	1.13	1.05	0.96	1.20	1.12	1.20	1.02	1.53	0.86	0.89	0.87	1.13	0.91	1.16	1.11	1.19	0.00	0.78	0.00
mTSS2	0.28	0.80	1.12	0.86	1.08	1.18	0.85	1.33	1.07	1.20	1.09	1.89	0.86	1.00	0.75	1.02	0.94	1.49	0.83	1.59	0.00	0.42	0.00
mTSS3	0.07	0.77	1.07	0.79	1.00	1.09	0.87	1.53	1.17	1.16	1.12	1.73	0.77	1.19	0.62	0.86	0.81	1.44	0.80	1.45	0.00	0.86	0.03
	100.0	7.16	6.67	5.86	5.73	5.56	5.48	5.33	4.74	4.56	4.79	4.47	4.40	4.41	4.57	3.81	3.60	3.48	3.32	2.25	0.00	6.26	3.36

B

States in the top 1 most enriched in with non-primary chromosomes

State	Genome %	chr1_GL456210_random	chr1_GL456211_random	chr1_GL456212_random	chr1_GL456213_random	chr1_GL456221_random	chr4_GL456216_random	chr4_GL456350_random	chr4_JH584292_random	chr4_JH584293_random	chr4_JH584294_random	chr4_JH584295_random	chr5_GL456354_random	chr5_JH584296_random	chr5_JH584297_random	chr5_JH584298_random	chr5_JH584299_random	chr7_GL456219_random	chrM
mGapArtf1	15	2.9	3.4	3.7	3	3.6	1.1	6.6	2.6	6.6	6.3	0	6.1	6.4	6.3	6.6	5.7	6.6	1.9
mGapArtf3	0	0	4	19	0	0	208	0	226	0	0	1591	0	0	0	0	0	0	29
mHET4	0.5	0	0	0	0	0	9.7	0	0	0	0	0	0	0	0	0	0	0	75
mHET7	0.2	19	38	43	0	24	4.5	0	6.7	0	0	0	0	0	0	0	0.2	0	0
mHET8	1.1	16	11	9.7	0	11	1.1	0	7.4	0	0	0	1.4	0.5	0.3	0	3.5	0	0
mHET9	2.8	7.7	6.6	4.8	0	7.5	1.6	0	11	0	0	0	2	0.9	1.4	0	3.3	0	0
mHET10	1.8	0.3	0.2	0.1	0	0.1	1.5	0	1.5	0	0	0	0.1	0	0	0	0	0	0
mZNF1	0.2	0	0	0	0	0	20	0	0	0	0	0	0	0	0	0	0	0	0
mEnhWk13	0.9	0	0	0	5.1	0	0.7	0	0	0	0	0	0	0	0	0	0	0	0

State	chrUn_GL456239	chrUn_GL456359	chrUn_GL456360	chrUn_GL456366	chrUn_GL456367	chrUn_GL456368	chrUn_GL456370	chrUn_GL456372	chrUn_GL456378	chrUn_GL456379	chrUn_GL456381	chrUn_GL456382	chrUn_GL456383	chrUn_GL456385	chrUn_GL456387	chrUn_GL456389	chrUn_GL456390	chrUn_GL456392	chrUn_GL456393	chrUn_GL456394	chrUn_GL456396	chrUn_JH584304	chrX_GL456233_random	chrY_JH584300_random	chrY_JH584301_random	chrY_JH584302_random	chrY_JH584303_random
mGapArtf1	0.2	1.6	1.7	2.3	2	2.1	2.1	2.4	1.6	2.8	1.2	3.5	1.2	2	0.8	1.1	2.7	0.2	0.8	1	0.3	0	2.4	6.6	6.6	6.6	6.6
mGapArtf3	597	147	0	0	11	0	341	0	121	0	0	0	383	0	679	217	466	222	172	79	225	1160	0	0	0	0	0
mHET4	0	10	3.6	2.4	0	0	1.4	0	0	0	0	0	0	0	0	0	0	0	0	0	0	0	0.5	0	0	0	0
mHET7	206	0	0	2.1	17	39	56	31	28	0	285	0	191	0	166	202	141	244	342	95	202	4.35	6.5	0	0	0	0
mHET8	7.8	8	0	2.3	16	9.9	3.4	20	3.5	13	1.4	9.5	7.6	21	0	7.7	0.7	2.3	2.6	17	16	0.16	12	0	0	0	0
mHET9	3.2	5.6	2.5	1.4	13	5.3	3.2	4.7	2.9	13	2.2	11	2	11	0.3	4.2	1.5	2.1	1.3	6.2	2.4	0	9.4	0	0	0	0
mHET10	0.3	3	11	18	0	3.9	1.7	0.4	3.6	2	0	0	0	0	0	0	0.5	1	0	0	0.5	0	0	0	0	0	0
mZNF1	0	0	0	0	0	0	0	0	0	0	0	0	0	0	0	0	0	0	0	0	0	0	20	0	0	0	0
mEnhWk13	0	0	0	0	0	0	0	0	0	0	0	0	0	0	0	0	0	0	0	0	0	0	0	0	0	0	0

Highest enr. across states

Supplementary Figure 3: Mouse full-stack states enrichments with different chromosomes. **(A)** The first and second columns show the mouse full-stack states and their percent genome coverage. The following columns correspond to different chromosomes. The heatmap shows fold enrichments of each state with each chromosome. Coloring of the heatmap is column-specific. The last row shows the percentage of the genome that each chromosome covers. Certain states in polycomb repressed group (mReprPC4-6) show distinctly high enrichments with chromosome X. **(B)** The first and second columns show mouse full-stack states and their genome coverage, respectively. The following columns correspond to different scaffold chromosomes. Only states that show highest enrichments with at least one scaffold chromosome are shown. Within each column, the highest enrichment values across 100 mouse full-stack states are colored red. States in ‘assembly gaps and alignment artifacts’ or in heterochromatin groups show highest enrichments with multiple scaffold chromosomes.

Enrichment of mouse full-stack states with classes of repeat elements

State	Genome %	DNA	DNA?	LINE	LINE?	LTR	LTR?	Low_complexity	Other	RC	RC?	RNA	SINE	SINE?	Satellite	Simple_repeat	Unknown	rRNA	scRNA	snRNA	srpRNA	tRNA
mGapArtf1	15.10	0.23	0.08	2.14	0.00	1.17	0.06	0.66	0.93	0.20	0.09	0.07	0.23	0.05	1.48	0.57	3.63	0.46	0.40	0.32	0.07	0.15
mGapArtf2	0.25	0.51	0.00	0.52	0.00	2.89	0.56	0.42	2.00	0.00	0.00	2.21	2.08	0.00	1.11	0.63	0.21	8.62	0.56	0.78	3.58	0.93
mGapArtf3	0.04	0.47	0.00	0.23	0.00	1.03	0.84	0.84	0.99	0.00	0.00	0.00	2.56	0.00	22.47	1.21	0.37	95.49	0.99	2.29	0.00	1.79
mQuies1	2.79	1.82	2.33	0.44	1.39	0.82	2.16	1.25	0.50	1.91	3.24	1.00	0.93	3.20	0.86	1.23	0.37	0.78	1.08	1.10	0.74	1.08
mQuies2	8.01	1.66	0.88	0.87	0.61	0.97	1.87	1.87	0.76	1.99	1.17	0.47	1.41	0.43	1.13	2.03	0.28	0.96	1.44	1.67	1.00	1.27
mQuies3	16.39	1.10	1.01	1.23	1.51	0.70	1.47	1.69	0.52	1.27	1.14	0.54	0.55	0.73	0.65	1.48	0.20	0.56	0.66	0.96	0.39	0.44
mHET1	0.81	0.53	0.13	1.04	0.00	1.81	0.27	0.26	17.53	0.51	0.00	2.55	0.79	0.00	2.30	1.20	0.11	1.62	0.56	0.35	1.44	1.05
mHET2	2.41	1.19	1.05	0.74	0.00	1.38	0.79	0.70	2.08	1.14	1.73	2.09	1.00	0.18	1.52	1.23	0.18	0.99	0.72	0.67	1.18	0.74
mHET3	3.56	0.66	0.47	1.64	0.26	1.49	0.55	0.42	1.29	0.62	0.74	2.03	0.82	0.64	0.52	0.60	0.15	0.82	0.73	1.01	1.81	0.68
mHET4	0.52	0.72	0.54	1.24	0.00	1.57	0.38	0.31	0.90	1.75	0.00	3.87	1.10	1.75	0.72	0.55	0.29	0.80	0.80	0.71	3.13	0.50
mHET5	1.23	0.40	0.06	1.55	0.00	3.01	0.42	0.33	2.02	0.08	0.00	1.48	0.72	0.00	0.54	0.41	0.31	1.48	0.57	0.96	0.81	0.66
mHET6	0.74	0.27	0.07	1.49	0.00	3.17	0.14	0.23	1.55	0.04	0.00	0.64	0.59	0.00	1.37	0.29	2.31	3.39	0.57	0.75	0.00	0.83
mHET7	0.20	0.23	0.00	1.06	0.00	3.25	0.64	0.21	1.51	0.00	0.00	3.88	0.72	0.00	7.56	0.41	4.91	9.91	0.49	0.43	3.07	2.05
mHET8	1.10	0.29	0.00	2.07	0.00	2.24	0.01	0.40	0.75	0.00	0.00	0.15	0.55	0.00	1.21	0.48	2.72	2.23	0.53	1.21	0.27	0.60
mHET9	2.80	0.32	0.07	2.72	0.00	1.48	0.09	0.54	0.63	0.12	0.00	0.46	0.44	0.12	0.92	0.58	1.53	0.48	0.52	1.24	0.38	0.38
mHET10	1.77	0.59	0.21	1.83	0.59	2.35	0.53	0.70	0.58	0.73	0.00	0.40	0.53	0.41	0.50	0.66	0.71	0.44	0.54	0.93	1.00	0.48
mZNF1	0.24	0.65	0.00	1.08	0.00	2.30	0.30	0.38	0.40	0.00	0.00	1.14	1.51	0.00	4.16	0.55	14.29	0.49	0.72	1.01	1.25	0.83
mZNF2	0.13	0.60	0.85	0.62	0.00	1.99	0.40	0.24	0.94	0.00	0.00	0.88	1.84	0.00	19.89	0.36	35.11	3.10	0.81	0.63	2.83	1.25
mReprPC1	0.76	1.04	0.49	0.28	0.00	1.04	0.40	0.71	1.51	0.19	0.00	2.91	1.79	0.00	1.29	0.79	0.35	1.61	1.47	0.78	1.19	1.69
mReprPC2	2.65	1.25	0.26	0.51	0.00	1.36	0.65	0.69	1.46	0.77	0.61	2.74	1.85	0.07	1.28	1.04	0.34	1.07	1.47	1.42	1.67	1.59
mReprPC3	0.94	1.21	1.52	0.26	1.16	0.86	1.41	0.84	0.97	2.09	2.58	1.15	1.17	1.76	1.39	0.91	0.25	0.82	0.79	0.54	1.00	0.68
mReprPC4	0.10	1.19	0.00	0.39	0.00	0.61	1.25	0.44	0.66	0.00	0.00	1.64	2.11	0.00	0.35	0.44	0.24	0.38	1.98	1.16	0.90	1.43
mReprPC5	1.21	1.68	1.73	0.89	0.83	0.97	1.38	1.49	0.99	1.63	0.00	2.36	1.36	1.18	1.20	1.26	0.36	1.05	1.33	1.12	0.90	1.27
mReprPC6	0.49	1.53	0.67	0.54	0.48	0.99	1.85	0.88	1.64	0.58	5.94	1.74	1.80	0.93	0.93	0.77	0.45	2.38	1.39	1.71	3.21	1.44
mReprPC_openC1	0.15	0.61	3.66	0.08	0.00	0.19	1.31	0.55	0.21	0.00	0.00	0.00	0.62	0.00	0.31	0.44	0.20	0.31	0.96	0.21	0.00	0.69
mReprPC_openC2	0.38	0.97	2.30	0.14	16.59	0.50	0.82	0.67	0.97	0.13	0.00	1.32	1.06	1.54	0.95	0.67	0.24	0.43	1.04	0.15	1.64	0.97
mReprPC_openC3	0.22	0.74	0.00	0.11	0.00	0.42	0.00	0.53	0.89	0.00	0.00	0.08	1.34	0.00	0.70	0.55	0.27	1.24	1.22	0.98	0.31	1.65
mReprPC_openC4	0.22	0.65	1.66	0.10	0.00	0.25	0.57	0.68	0.22	0.00	0.00	0.77	1.14	2.73	0.73	0.78	0.37	0.67	1.57	1.60	0.58	1.84
mOpenC1	0.46	0.41	0.58	0.10	0.00	0.52	0.06	0.26	5.60	0.37	0.00	2.45	1.04	0.00	0.47	0.41	0.11	2.37	0.94	0.65	2.18	0.66
mOpenC2	1.15	0.82	0.23	0.24	0.00	1.00	0.62	0.37	5.24	0.33	0.00	2.71	1.75	0.00	0.75	0.75	0.22	1.57	1.34	0.74	1.08	1.45
mOpenC3	0.43	1.41	1.53	0.19	0.00	0.73	2.25	0.42	0.88	2.33	5.92	1.06	0.93	2.20	0.36	0.59	0.40	1.25	0.84	0.34	1.26	1.02
mOpenC4	0.90	1.83	2.04	0.30	0.00	0.80	1.74	0.77	0.63	2.10	2.35	1.68	1.00	5.09	0.62	0.79	0.42	0.86	1.06	0.84	2.15	1.22
mOpenC5	0.24	0.61	0.23	0.10	0.00	0.49	0.67	0.48	2.31	2.07	0.00	1.33	1.06	0.79	0.81	0.58	0.20	0.76	0.87	0.57	1.74	2.18
mOpenC6	0.14	0.76	0.00	0.08	0.00	0.25	0.34	0.54	0.83	0.00	0.00	2.56	0.92	0.26	0.73	0.62	0.28	6.47	0.56	5.64	4.24	5.47
mOpenC7	0.20	1.10	0.00	0.17	0.00	0.50	0.03	0.90	1.16	1.85	0.00	0.81	2.40	0.00	0.87	0.93	0.36	4.79	0.98	0.73	0.96	1.34
mOpenC8	0.22	1.29	0.78	0.26	0.00	0.82	0.15	1.18	7.07	0.00	2.92	0.82	1.91	2.12	4.42	2.40	0.31	7.70	1.47	6.01	4.05	4.15
mOpenC9	1.38	1.63	1.63	0.39	3.58	0.69	2.44	0.74	0.41	2.46	4.70	1.69	0.83	4.83	0.53	0.84	0.37	1.05	0.69	1.11	0.60	0.82
mEnhA1	0.26	1.22	2.28	0.13	9.39	0.34	3.64	0.37	0.39	4.70	0.00	1.11	0.52	4.21	0.32	0.46	0.72	0.38	0.84	0.57	0.00	1.09
mEnhA2	0.24	0.69	3.26	0.07	0.00	0.19	1.34	0.46	0.20	0.00	0.00	0.65	0.51	3.38	0.31	0.43	0.23	0.00	0.54	0.14	0.97	0.86
mEnhA3	0.15	0.46	4.19	0.05	0.00	0.13	0.71	0.40	0.06	2.15	0.00	0.64	0.52	0.00	0.31	0.38	0.13	0.36	0.63	0.47	0.00	0.95
mEnhA4	0.28	0.84	1.84	0.09	0.00	0.27	2.10	0.42	0.13	0.12	0.00	0.69	0.71	1.26	0.36	0.42	0.21	0.23	0.47	0.33	1.40	0.55
mEnhA5	0.28	0.97	0.84	0.12	0.00	0.59	0.28	0.44	0.71	1.08	0.59	0.88	1.19	0.63	0.61	0.52	0.26	0.97	1.71	0.44	0.27	1.51
mEnhA6	0.40	1.55	2.21	0.20	0.00	0.44	1.26	0.80	0.19	1.76	2.81	0.61	1.16	2.44	0.95	0.72	0.34	1.11	1.87	1.03	1.55	1.71
mEnhA7	0.38	0.96	3.14	0.11	9.47	0.40	1.88	0.64	0.25	3.34	1.17	0.47	0.77	4.18	0.61	0.57	0.32	1.06	0.57	0.83	2.48	0.79
mEnhA8	0.67	1.28	6.40	0.21	12.61	0.52	2.19	0.89	0.20	4.15	0.60	1.71	0.82	4.50	1.06	0.89	0.39	0.73	0.82	0.53	2.89	1.22
mEnhA9	0.36	1.06	9.95	0.12	11.26	0.29	4.15	0.57	0.16	2.60	2.04	1.68	0.51	5.63	0.74	0.63	1.15	0.82	0.49	0.24	1.57	0.82
mEnhA10	0.20	0.69	5.02	0.07	0.00	0.18	2.41	0.61	0.06	0.83	6.60	0.36	0.38	6.04	0.35	0.53	0.87	1.06	0.67	0.00	0.00	0.32
mEnhA11	0.13	0.56	3.01	0.06	0.00	0.17	0.16	0.72	0.04	0.00	0.00	0.00	0.44	11.60	0.25	0.51	0.20	0.20	0.90	0.97	0.75	0.67
mEnhA12	0.18	0.66	0.00	0.09	0.00	0.35	2.08	0.42	0.38	1.04	0.00	1.95	1.09	0.00	0.49	0.40	0.39	1.40	1.22	0.29	0.85	0.66
mEnhA13	0.28	1.04	0.47	0.17	0.00	0.72	0.88	0.53	0.76	0.67	0.00	1.49	1.29	0.00	0.49	0.65	0.17	1.76	2.06	0.49	4.76	1.39
mEnhA14	0.46	1.14	0.00	0.22	0.00	0.91	0.51	0.62	1.16	0.53	0.97	1.51	1.86	0.76	1.10	1.02	0.30	2.21	1.45	1.05	1.61	2.17
mEnhA15	0.34	1.00	0.46	0.16	0.00	0.50	1.37	0.49	0.70	0.00	0.00	1.66	1.14	2.24	0.72	0.60	0.28	0.32	1.15	0.89	1.45	1.73
mEnhA16	0.40	1.17	5.04	0.13	8.37	0.46	0.95	0.58	0.38	1.30	0.00	1.28	0.75	7.63	0.63	0.55	0.48	0.05	0.47	0.43	0.94	0.98
mEnhA17	0.23	1.08	0.00	0.18	0.00	0.63	0.26	0.57	1.31	3.02	0.00	0.71	1.73	0.74	0.57	0.73	0.31	2.26	1.80	0.70	0.35	3.01
mEnhA18	0.31	1.53	0.00	0.37	0.00	0.96	1.04	1.17	1.03	1.35	0.00	2.02	2.19	0.00	1.07	1.25	0.35	0.91	2.34	1.49	0.75	1.82
mEnhWk1	0.80	0.92	0.47	0.41	2.33	2.38	0.95	0.98	0.68	0.54	0.00	1.09	1.89	1.29	0.92	1.00	0.42	0.98	1.92	1.14	0.59	1.37
mEnhWk2	0.45	0.90	0.92	0.20	0.00	1.37	0.59	0.86	1.80	0.25	2.10	1.99	1.56	0.00	0.55	0.75	0.29	1.94	1.41	0.58	3.69	1.37
mEnhWk3	0.47	1.11	1.72	0.16	0.00	0.52	3.31	0.30	0.72	3.02	0.00	1.73	0.83	0.82	0.46	0.49	0.56	1.17	0.63	0.39	0.64	0.79
mEnhWk4	0.41	1.29	1.05	0.19	0.00	0.91	1.19	0.64	0.69	1.58	4.99	2.69	1.67	0.52	0.94	0.85	0.30	1.59	1.61	0.38	1.43	1.93
mEnhWk5	0.87	1.49	0.85	0.35	2.52	1.24	1.25	1.00	0.71	2.86	0.94	0.76	1.68	0.00	0.99	1.33	0.41	1.34	1.58	1.23	2.12	1.72
mEnhWk6	0.42	1.58	0.36	0.32	1.73	0.96	1.13	1.07	0.46	0.73	0.00	1.57	1.38	1.15	0.98	1.02	0.37	2.61	1.78	1.24	2.08	1.52
mEnhWk7	1.37	1.59	0.23	0.46	0.35	1.08	0.84	1.29	0.78	1.04	1.16	0.70	1.63	0.57	1.71	1.51	0.34	0.99	1.75	1.48	1.15	1.14
mEnhWk8	1.57	1.49	8.96	0.32	9.14	0.52	2.88	1.15	0.39	2.77	3.28	0.69	0.73	4.29	1.10	1.22	0.57	0.58	0.76	0.67	0.37	0.79
mEnhWk9	0.62	1.63	8.46	0.22	3.47	0.40	4.18	0.56	0.18	1.86	2.03	1.87	0.57	7.81	0.50	0.64	1.25	0.26	0.52	0.79	0.57	0.80
mEnhWk10	0.73	1.47	1.08	0.34	3.14	0.79	2.16	0.91	0.59	1.28	0.00	1.83	1.29	1.25	1.18	0.96	0.30	1.41	1.33	0.90	1.90	1.43
mEnhWk11	0.92	1.59	4.56	0.23	5.35	0.68	2.21	0.86	0.33	1.82	5.27	1.17	0.91	11.64	0.96	0.81	0.57	0.43	0.88	0.59	0.42	1.01
mEnhWk12	0.35	1.07	0.23	0.22	0.00	1.10	0.77	0.64	2.31	0.38	0.00	2.07	2.11	0.56	0.96	0.80	0.49	2.12	2.03	1.27	1.81	1.62
mEnhWk13	0.90	1.54	0.95	0.53	0.00	1.20	1.04	1.37	0.85	0.99	3.68	1.92	1.85	1.09	0.97	1.36	0.34	0.92	1.84	1.56	1.66	1.75
mTxEnh1	0.24	0.91	0.00	0.13	0.00	0.38	0.15	0.24	0.83	0.25	3.83	1.51	1.44	1.94	0.45	0.39	0.31	1.63	1.54	0.58	3.40	1.57
mTxEnh2	0.36	1.45	0.72	0.25	0.00	0.56	0.69	0.52	0.64	2.25	1.46	2.34	2.48	1.20	0.38	0.59	0.31	1.42	2.74	1.32	2.55	2.77
mTxEnh3	0.75	1.80	0.50	0.39	0.00	0.60	0.55	0.94	0.48	1.20	1.08	1.64	2.42	1.16	0.46	0.92	0.34	0.75	2.44	2.39	2.23	2.37
mTxEnh4	0.25	0.83	0.00	0.14	0.00	0.52	0.56	0.23	0.47	0.00	0.00	1.87	2.29	0.00	0.23	0.29	0.32	0.92	2.28	1.48	2.40	2.83
mTxEnh5	0.37	0.57	0.17	0.10	0.00	0.42	0.71	0.25	1.82	0.50	0.00	1.93	1.22	0.61	0.53	0.35	0.14	1.12	0.83	0.36	2.19	1.76
mTxEnh6	0.19	0.79	3.11	0.11	0.00	0.29	0.52	0.25	0.34	0.00	0.00	0.00	0.95	0.27	0.47	0.40	0.28	0.76	0.78	0.00	0.00	0.99
mTx1	0.96	1.43	0.31	0.48	0.00	0.51	0.83	1.55	0.30	0.00	0.19	0.69	2.01	0.39	0.61	1.14	1.09	0.52	2.18	1.85	1.05	1.75
mTx2	1.07	1.23	0.30	0.37	0.00	0.60	0.47	0.88	0.37	0.49	1.31	1.60	2.52	0.44	0.75	0.80	0.54	0.68	2.66	2.35	2.23	2.48
mTx3	0.96	1.16	0.03	0.25	0.00	0.54	0.70	0.63	0.32	0.24	2.47	1.26	2.73	0.86	0.60	0.51	0.61	1.50	2.46	2.19	2.44	2.11
mTx4	0.91	0.77	0.00	0.14	0.00	0.49	0.31	0.24	0.37	0.51	0.00	2.78	2.44	0.33	0.69	0.26	0.66	0.78	2.12	1.31	2.74	2.30
mTx5	0.62	0.74	0.16	0.16	0.00	0.57	0.00	0.29	0.93	0.56	0.00	1.95	1.86	0.00	0.70	0.47	0.30	1.03	1.51	0.92	1.24	1.56
mTx6	0.44	1.18	1.29	0.27	0.00	0.73	0.89	0.53	0.47	0.94	0.00	1.47	2.64	1.79	0.33	0.57	0.45	1.37	2.26	2.30	3.80	2.44
mTx7	0.68	1.01	0.56	0.21	0.00	0.67	0.55	0.38	1.16	0.29	2.83	0.93	1.87	0.00	0.62	0.70	0.21	1.48	1.61	1.07	1.77	2.22
mTx8	0.35	0.91	0.46	0.22	0.00	0.56	0.52	0.36	0.48	1.17	0.00	2.38	1.45	0.00	1.29	0.64	0.39	0.64	1.23	0.98	1.06	1.21
mTxEx1	0.46	0.28	0.09	0.05	0.00	0.23	0.00	0.14	0.86	0.00	0.00	2.73	0.96	0.00	0.36	0.19	0.10	0.54	0.59	0.74	1.96	0.81
mTxEx2	0.18	0.34	0.35	0.04	0.00	0.16	0.39	0.17	0.68	0.64	0.00	2.60	0.87	0.00	0.41	0.20	0.09	0.25	0.37	0.00	2.19	0.77
mTxEx3	0.04	0.31	0.00	0.04	0.00	0.15	0.00	0.27	0.04	0.00	0.00	2.27	1.22	0.00	8.11	0.23	0.13	49.46	0.89	4.23	6.66	1.29
mTxWk1	2.32	1.58	0.39	0.71	0.00	0.55	0.84	1.98	0.32	0.20	0.85	0.10	1.51	0.48	0.66	1.52	0.79	0.81	1.69	1.56	1.31	1.35
mTxWk2	1.23	1.33	0.93	0.51	0.00	0.83	0.52	0.64	0.51	0.92	1.14	0.87	1.83	0.19	0.82	0.87	0.63	0.92	1.55	1.26	1.65	1.65
mBivProm1	0.13	0.19	0.26	0.03	0.00	0.07	0.00	0.68	0.10	0.00	0.00	0.00	0.41	0.00	0.45	0.47	0.21	2.75	0.15	0.33	0.00	4.26
mBivProm2	0.14	0.10	0.18	0.01	0.00	0.01	0.28	1.84	0.07	0.00	0.00	0.00	0.10	4.22	0.11	0.50	0.06	3.28	0.20	0.47	0.00	1.66
mBivProm3	0.09	0.24	0.45	0.03	0.00	0.05	0.00	1.05	0.00	0.00	0.00	0.00	0.26	0.00	0.37	0.76	0.21	0.00	0.38	0.00	0.00	0.13
mPromF1	0.11	0.28	0.03	0.05	0.00	0.14	0.00	1.90	0.01	0.00	0.00	0.00	0.28	1.45	0.53	1.04	0.01	0.91	0.39	0.13	0.00	1.42
mPromF2	0.19	0.34	0.00	0.04	0.00	0.11	0.00	0.50	0.05	0.00	0.00	0.00	0.83	1.76	0.18	0.34	0.07	0.33	0.70	1.30	0.00	1.16
mPromF3	0.14	0.42	0.68	0.06	0.00	0.21	1.21	0.53	0.07	0.96	0.00	1.05	0.88	0.00	0.46	0.45	0.21	0.23	0.48	0.47	0.79	2.15
mPromF4	0.14	0.85	2.49	0.10	0.00	0.37	0.32	1.35	0.14	0.00	0.00	0.00	0.92	6.32	0.92	1.13	0.43	2.60	1.25	0.78	0.00	4.06
mPromF5	0.14	0.91	0.00	0.08	0.00	0.19	0.00	0.67	0.05	1.70	0.00	0.52	1.65	0.00	0.20	0.55	0.32	0.77	1.87	1.68	1.82	0.99
mPromF6	0.13	1.27	0.00	0.14	0.00	0.30	0.00	0.62	0.13	0.19	0.00	2.20	2.55	0.00	0.18	0.57	0.52	0.90	2.53	1.18	6.92	3.07
mPromF7	0.14	0.78	0.38	0.12	0.00	0.22	0.00	1.41	0.03	0.00	0.00	0.00	0.77	0.00	0.60	1.23	0.10	0.95	1.75	0.57	0.94	0.83
mTSS1	0.15	0.07	0.00	0.01	0.00	0.04	0.00	3.59	0.01	0.00	0.00	0.00	0.06	0.00	0.15	0.83	0.00	5.20	0.19	0.00	0.00	0.38
mTSS2	0.28	0.08	0.14	0.01	0.00	0.03	0.00	3.75	0.01	0.00	0.00	0.00	0.16	0.00	0.08	0.83	0.04	1.06	0.36	0.96	0.00	3.18
mTSS3	0.07	0.26	1.07	0.03	0.00	0.09	0.00	3.02	0.00	0.00	0.00	0.00	0.40	0.00	0.27	1.09	0.01	0.63	2.54	13.79	13.90	54.07
	100.00	1.08	0.01	20.08	0.0003	11.68	0.01	0.75	0.30	0.002	0.0004	0.004	7.48	0.001	0.19	2.17	0.10	0.01	0.02	0.01	0.002	0.01

Supplementary Figure 4: Mouse full-stack states enrichments with different classes of repeats (Smit *et al.*, 2015). The first and second columns show the mouse full-stack states and their genome coverage. The following columns correspond to different repeat classes. The following columns correspond to different classes of repeat elements. The heatmap shows fold enrichments of each state with each repeat class. Coloring of the heatmap is column-specific. The last row shows the percentage of the genome that each repeat class covers.

Top mouse full-stack states most enriched with classes of repeat elements

State	Genome %	snRNA	srpRNA	tRNA	Low_complexity	SINE	scRNA	RC	Simple_repeat	DNA	Unknown	LINE	LTR	RNA	Other	Satellite	rRNA
mGapArtf3	0.04	2.29	0.00	1.79	0.84	2.56	0.99	0.00	1.21	0.47	0.37	0.23	1.03	0.00	0.99	22.47	95.49
mHET1	0.81	0.35	1.44	1.05	0.26	0.79	0.56	0.51	1.20	0.53	0.11	1.04	1.81	2.55	17.53	2.30	1.62
mHET7	0.20	0.43	3.07	2.05	0.21	0.72	0.49	0.00	0.41	0.23	4.91	1.06	3.25	3.88	1.51	7.56	9.91
mHET9	2.80	1.24	0.38	0.38	0.54	0.44	0.52	0.12	0.58	0.32	1.53	2.72	1.48	0.46	0.63	0.92	0.48
mZNF2	0.13	0.63	2.83	1.25	0.24	1.84	0.81	0.00	0.36	0.60	35.11	0.62	1.99	0.88	0.94	19.89	3.10
mOpenC4	0.90	0.84	2.15	1.22	0.77	1.00	1.06	2.10	0.79	1.83	0.42	0.30	0.80	1.68	0.63	0.62	0.86
mOpenC8	0.22	6.01	4.05	4.15	1.18	1.91	1.47	0.00	2.40	1.29	0.31	0.26	0.82	0.82	7.07	4.42	7.70
mEnhA1	0.26	0.57	0.00	1.09	0.37	0.52	0.84	4.70	0.46	1.22	0.72	0.13	0.34	1.11	0.39	0.32	0.38
mTxEnh2	0.36	1.32	2.55	2.77	0.52	2.48	2.74	2.25	0.59	1.45	0.31	0.25	0.56	2.34	0.64	0.38	1.42
mTx3	0.96	2.19	2.44	2.11	0.63	2.73	2.46	0.24	0.51	1.16	0.61	0.25	0.54	1.26	0.32	0.60	1.50
mTSS2	0.28	0.96	0.00	3.18	3.75	0.16	0.36	0.00	0.83	0.08	0.04	0.01	0.03	0.00	0.01	0.08	1.06
mTSS3	0.07	13.79	13.90	54.07	3.02	0.40	2.54	0.00	1.09	0.26	0.01	0.03	0.09	0.00	0.00	0.27	0.63

Highest enr. across states

Supplementary Figure 5: Enrichment of select mouse full-stack states with different classes of repeat elements (Smit *et al.*, 2015). The first and second columns show mouse full-stack states and their genome coverage, respectively. The following columns correspond to different classes of select repeat elements (with elements named with '?' excluded). *Only states that show highest enrichments with at least one repeat class are shown*. Within each column, the highest enrichment values across 100 mouse full-stack states are colored red.

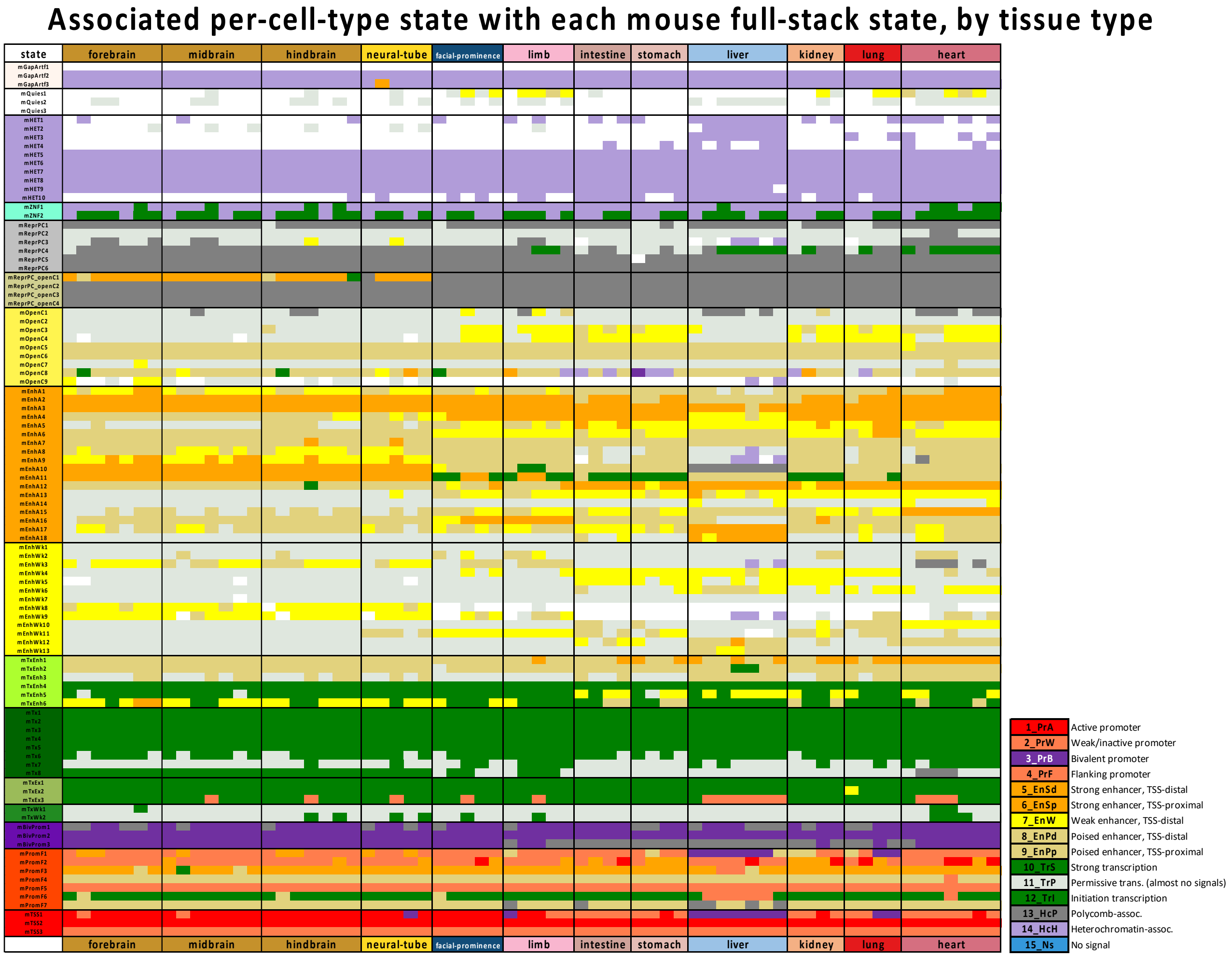

Supplementary Figure 6: Full-stack states maximum-enrichments with annotated concatenated-model chromatin states in 66 mouse reference epigenomes (Gorkin *et al.*, 2020)**.** Each row corresponds to one of 100 mouse full-stack state (**Methods**). Each column corresponds to a reference epigenome, grouped by the associated cell types as colored at the top and bottom. Each cell corresponds to a reference epigenome and mouse full-stack state combination. The cell's color corresponds to the chromatin state from the concatenated 15-state model that is most enriched with the respective mouse full-stack state. Description of states in the per-cell-type 15-state concatenated model is in the bottom (Gorkin et al., 2020). The figure highlights how some mouse full-stack states are maximally enriched with the same concatenated-model chromatin states across all the reference epigenomes; for example, states mTx1-5 are maximally enriched with the strong transcription state in all 66 reference epigenomes' 15-state concatenated annotation. Other mouse full-stack states are enriched for distinct concatenated states in different cell types, for example state mEnhA17-- characterized as an enhancer state in liver, spleen and bone marrow based on emission probabilities of enhancer associated marks-- is most enriched with an active enhancer in liver cell types, while being most enriched with poised/weak enhancer states in others. Fully-annotated, detailed description of each mouse full-stack state enrichment patterns with concatenated per-cell-type states can be found in **Additional File 5**.

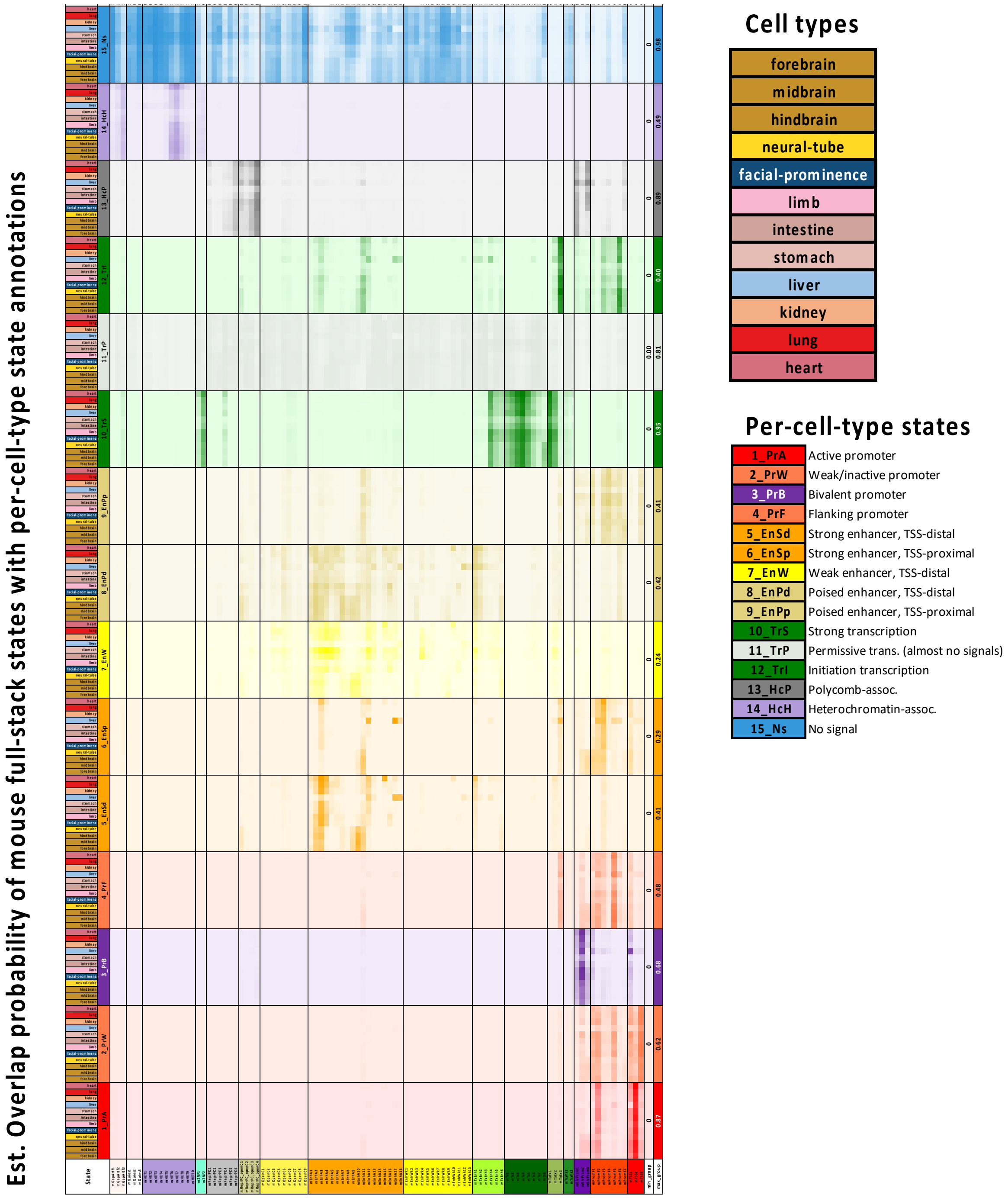

Supplementary Figure 7: Estimated probabilities of per-cell-type concatenated-model chromatin states overlapping with mouse full-stack states. The figure shows estimated probabilities of per-cell-type chromatin state annotations overlapping with mouse full-stack states observed in different cell groups (Gorkin *et al.*, 2020). A fully-annotated version of this figure is also provided as an excel file in **Additional File 5**. The figure is based on a 15-state per-cell type chromatin state model trained on 66 mouse reference epigenomes from 12 cell groups (Gorkin *et al.*, 2020). Each row corresponds to a combination of per-cell type state (among 15 states) and cell

group, as denoted in the first two columns and legends on the right and matching with the colors in **Fig. S6**. We note that we changed here the concatenated-model no-signal state from white to blue for better visibility. Rows corresponding to the same per-cell-type model state are grouped together (into 15 bigger rows). The 100 following columns correspond to 100 mouse full-stack states. Values in the heatmap correspond to the estimated probability a genomic position annotated as a mouse full-stack state (column) is also annotated as a concatenated-model state in a reference epigenome from the corresponding cell group (row) (**Methods**). The last two columns show the minimum and maximum probabilities observed for each per-cell type state for any combination of tissue group and mouse full-stack state. The heatmap colors correspond to the 15-state's colors and are scaled such that the maximum probability value in each row block is colored darkest (as seen in the right most column). The figure complements **Fig. S6** in providing information on how each full-stack state can correspond to different per-cell-type states, hence stratifying mouse full-stack states' characteristics in more details. For example, mouse full-stack state mTSS1 shows high probabilities of overlapping bivalent promoter state in liver cells, and moderate probabilities of overlapping the flanking/weak promoter state in other cell groups.

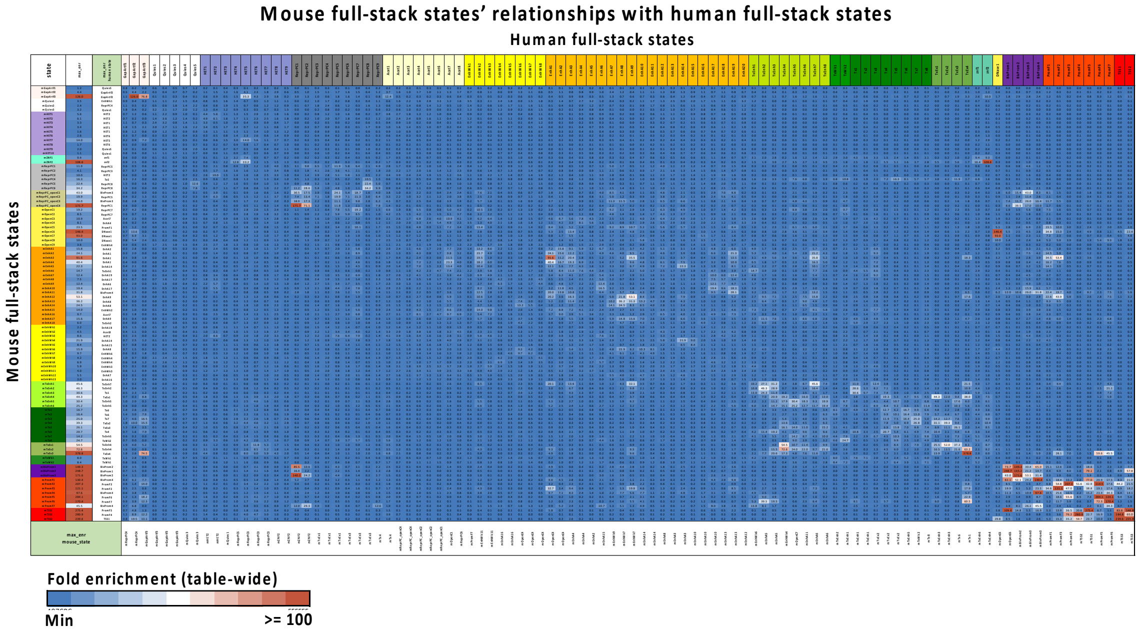

Supplementary Figure 8: Enrichments of mouse full-stack states with human full-stack states (Vu and Ernst, 2022). The first three columns show mouse full-stack states, the maximum fold enrichment with a human full-stack state (across all human states) and the corresponding human state, respectively. The following columns show the overlap enrichments with of each mouse state (rows) with each human state (columns). Across all pairs of states, the smallest enrichment values are colored blue and enrichment values >= 100 are colored red. The last row shows the mouse states that were maximally enriched with each human state, compared to other 99 mouse states. Thirty-eight out of 100 mouse full-stack states were one-to-one mapped to a human state, with the fold-enrichment ranging from 3.21 (mQuies2 and human Quies1 states) to 378.33 (mTxEx3 and human TxEx4 states). We note that 65 unique human states were maximally enriched with at least one mouse state and conversely, 59 unique mouse states were the maximally state for at least one human state. This figure is also provided in **Additional File 4.**

Mouse full-stack states' relationships with functional assay conservation (LECIF), sequence conservation (PhastCons) and human full-stack states

A

State	avg_lecif	Enr. With phastConsEle60w	Enr. With human state	Most enr. Human state	is 1 to 1 with human state
mGapArtf1	0.05	0.34	1.23	Quies5	
mGapArtf2	0.10	0.72	4.42	GapArtf2	
mGapArtf3	0.18	1.62	125.97	GapArtf2	*
mQuies1	0.05	1.25	3.50	EnhWk1	
mQuies2	0.05	0.76	2.40	ReprPC4	
mQuies3	0.05	0.70	3.21	Quies1	*
mHET1	0.08	0.82	5.62	HET2	
mHET2	0.06	0.65	6.08	HET2	
mHET3	0.05	0.47	3.32	HET1	
mHET4	0.05	0.76	3.41	HET1	
mHET5	0.06	0.42	1.60	HET1	
mHET6	0.06	0.52	2.60	HET6	
mHET7	0.08	0.75	14.00	HET5	
mHET8	0.05	0.45	1.70	HET6	
mHET9	0.04	0.37	1.99	Quies5	
mHET10	0.04	0.38	1.14	Quies4	
mZNF1	0.15	0.91	9.40	znf1	
mZNF2	0.19	1.85	108.64	znf2	*
mReprPC1	0.21	0.77	11.92	ReprPC5	
mReprPC2	0.08	0.64	4.14	ReprPC6	
mReprPC3	0.11	0.94	10.02	HET2	*
mReprPC4	0.16	2.37	16.32	Tx5	*
mReprPC5	0.08	1.06	22.39	ReprPC8	
mReprPC6	0.22	1.17	34.15	ReprPC8	*
mReprPC_openC1	0.41	2.49	43.00	BivProm3	
mReprPC_openC2	0.24	1.36	19.89	ReprPC5	
mReprPC_openC3	0.38	1.74	25.99	BivProm3	
mReprPC_openC4	0.60	1.90	171.74	ReprPC1	*
mOpenC1	0.27	1.64	19.20	ReprPC7	*
mOpenC2	0.14	0.95	8.50	ReprPC7	
mOpenC3	0.12	1.72	10.86	Acet7	
mOpenC4	0.08	1.54	8.15	EnhA4	*
mOpenC5	0.28	1.47	23.50	PromF1	
mOpenC6	0.43	1.51	146.36	DNase1	*
mOpenC7	0.26	1.18	93.01	DNase1	
mOpenC8	0.12	1.05	9.97	DNase1	
mOpenC9	0.06	1.58	3.62	EnhWk4	
mEnhA1	0.20	2.78	15.84	EnhA2	
mEnhA2	0.36	2.68	24.06	EnhA1	
mEnhA3	0.53	2.63	91.61	EnhA1	*
mEnhA4	0.34	2.14	40.42	EnhA1	
mEnhA5	0.26	1.15	22.28	EnhA14	*
mEnhA6	0.19	1.64	14.66	TxEnh1	*
mEnhA7	0.21	2.35	12.37	EnhA19	
mEnhA8	0.12	2.53	7.50	EnhA17	
mEnhA9	0.20	4.02	12.38	EnhA6	
mEnhA10	0.35	4.44	19.36	EnhA17	*
mEnhA11	0.45	4.00	31.83	BivProm4	
mEnhA12	0.43	1.54	53.11	EnhA9	*
mEnhA13	0.27	1.09	36.20	EnhA8	*
mEnhA14	0.16	0.76	24.46	EnhA8	
mEnhA15	0.21	1.37	13.98	EnhWk2	
mEnhA16	0.17	2.42	9.73	Acet7	
mEnhA17	0.27	1.04	15.61	EnhA9	
mEnhA18	0.15	0.77	5.99	TxEnh2	
mEnhWk1	0.06	0.61	2.17	EnhA18	
mEnhWk2	0.11	0.87	4.53	Acet8	
mEnhWk3	0.16	1.54	8.64	HET2	
mEnhWk4	0.15	0.87	21.92	EnhA14	
mEnhWk5	0.07	0.75	8.39	EnhA15	
mEnhWk6	0.15	1.02	15.95	EnhA8	
mEnhWk7	0.08	0.75	9.74	EnhWk6	
mEnhWk8	0.07	2.38	4.17	EnhWk4	
mEnhWk9	0.09	3.50	6.91	EnhWk4	*
mEnhWk10	0.10	0.98	5.94	EnhWk3	
mEnhWk11	0.08	2.27	4.01	EnhWk3	
mEnhWk12	0.14	0.73	5.54	EnhA7	
mEnhWk13	0.07	0.73	2.99	EnhA16	
mTxEnh1	0.47	1.07	45.64	TxEnh7	*
mTxEnh2	0.44	0.87	46.25	TxEnh2	*
mTxEnh3	0.26	0.90	30.61	Tx1	*
mTxEnh4	0.35	1.81	44.30	TxEx1	*
mTxEnh5	0.35	2.13	30.36	TxEnh5	*
mTxEnh6	0.34	2.44	25.24	TxEnh5	
mTx1	0.23	1.53	16.73	Tx6	*
mTx2	0.24	1.55	16.38	Tx6	
mTx3	0.25	1.81	25.03	Tx7	
mTx4	0.30	2.54	39.28	TxEx2	
mTx5	0.29	1.99	26.06	TxEx3	
mTx6	0.27	1.16	20.74	Tx4	*
mTx7	0.22	1.36	14.22	TxEnh5	
mTx8	0.34	1.95	24.72	TxWk2	*
mTxEx1	0.39	4.06	54.46	TxEnh4	
mTxEx2	0.48	3.54	72.57	TxEnh4	*
mTxEx3	0.63	3.93	378.83	TxEx4	*
mTxWk1	0.14	1.19	7.95	TxWk1	
mTxWk2	0.15	1.18	8.41	TxWk1	*
mBivProm1	0.61	3.13	149.26	BivProm2	
mBivProm2	0.69	4.59	248.67	BivProm1	*
mBivProm3	0.71	3.79	171.56	BivProm2	*
mPromF1	0.57	4.20	130.43	BivProm4	*
mPromF2	0.60	2.26	207.34	PromF3	*
mPromF3	0.52	2.40	121.14	PromF2	*
mPromF4	0.40	2.28	97.56	BivProm4	
mPromF5	0.67	1.51	284.05	PromF6	*
mPromF6	0.63	1.29	170.37	PromF7	*
mPromF7	0.43	2.88	45.46	BivProm4	
mTSS1	0.65	4.63	275.41	PromF5	*
mTSS2	0.67	3.92	280.90	PromF4	*
mTSS3	0.63	3.16	239.61	TSS1	*

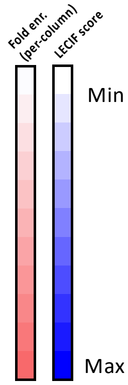

B

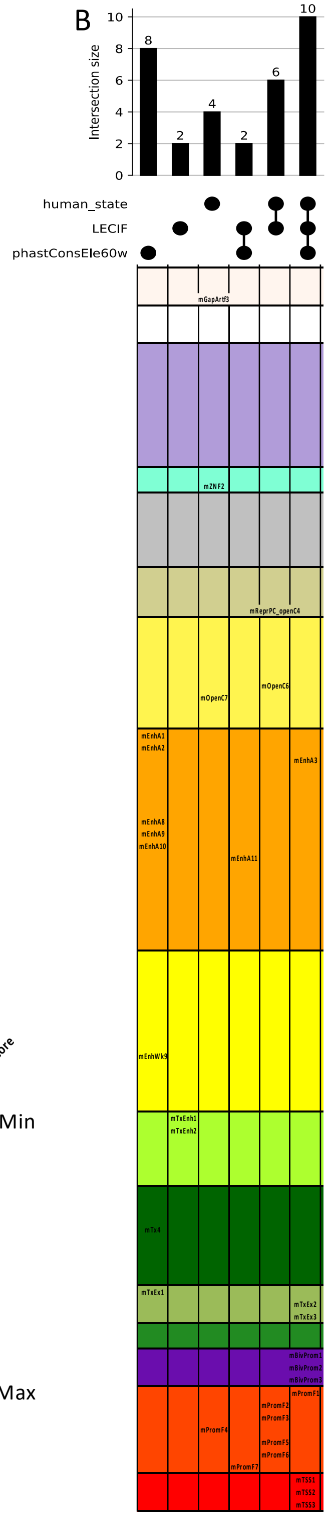

Supplementary Figure 9: Mouse full-stack states' relationship with LECIF scores, human full-stack states and phastCons elements. LECIF scores were developed to measure the level of evidence of human-mouse conservation at functional/epigenomic levels, with higher score (maximum of 1 and minimum of 0) implies higher evidence of conservation (Kwon and Ernst, 2021). PhastCons elements correspond to genomic regions showing strong 60-way multi-species sequence alignment conservation (Siepel *et al.*, 2005). Human full-stack states were learned from >1,000 Chip-seq/DNase-seq datasets in human, and provide annotation of the human genome that is shared across cell/tissue types (Vu and Ernst, 2022). **(A)** The heatmap shows mouse full-stack states (rows)' average LECIF scores, enrichments with phastCons elements and the maximum enrichments with human full-stack states. The first column shows the mouse full-stack states. Coloring of the next three columns is column specific, as specified in legend. The last two column shows the human state with the highest enrichment with each of the mouse state, and a star (*) next to a mouse state that is a one-to-one mapping with a human state. Here, one-to-one mapping of states means that the mouse state is most enriched with the human state compared to other 99 mouse states, and vice versa with the human state (**Methods**). **(B)** Upset plot showing the number of states that are among the top 20 states with either (1) highest average LECIF score, or (2) highest enrichments with PhastCons elements or (3) highest maximal enrichments with human full-stack states. Within each category, the column below the upset plot lists states that are in the top 20 most associated (as measured by average LECIF scores or fold enrichments) with the combination enrichment contexts.

A Neg. Log. Likelihood, AIC, BIC

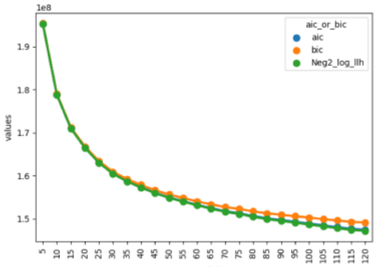

states (5 → 120)

B Correlations (in emission prob.) between 100-state models with other models

state	5	10	15	20	25	30	35	40	45	50	55	60	65	70	75	80	85	90	95	100	105	110	115	120
mGapArtf1	0.46	0.57	0.57	0.56	0.56	0.56	0.69	0.69	0.71	0.71	0.87	0.84	0.82	0.9	0.81	0.81	0.93	0.99	0.99	1	0.98	0.98	0.99	0.99
mGapArtf2	0.63	0.84	0.83	0.81	0.81	0.81	0.82	0.81	0.91	0.9	0.93	0.93	0.92	0.98	0.99	1	1	1	1	1	0.95	0.96	0.95	0.97
mGapArtf3	0.46	0.72	0.74	0.76	0.78	0.77	0.74	0.74	0.73	0.74	0.97	0.97	0.97	0.99	1	1	1	1	0.94	1	0.95	0.96	0.96	0.96
mQuies1	0.43	0.53	0.45	0.54	0.7	0.78	0.71	0.67	0.85	0.9	0.83	0.85	0.97	0.84	0.96	0.95	0.97	0.98	0.98	1	0.95	0.94	0.83	0.97
mQuies2	0.42	0.48	0.43	0.66	0.65	0.71	0.81	0.79	0.89	0.94	0.96	0.96	0.98	0.95	0.95	0.96	0.96	0.99	1	1	1	0.99	0.99	0.98
mQuies3	0.74	0.82	0.86	0.9	0.9	0.91	0.89	0.88	0.88	0.88	0.97	0.95	0.94	0.93	0.98	0.95	0.93	1	1	1	1	1	1	1
mHET1	0.7	0.64	0.84	0.77	0.76	0.73	0.81	0.79	0.84	0.84	0.95	0.89	0.95	0.96	0.93	0.95	0.96	0.94	0.91	1	0.98	0.96	0.98	0.96
mHET2	0.77	0.75	0.86	0.78	0.77	0.75	0.9	0.88	0.86	0.87	0.86	0.87	0.85	0.95	0.99	0.97	0.96	0.98	0.99	1	0.99	0.99	0.99	0.99
mHET3	0.83	0.87	0.79	0.87	0.88	0.89	0.93	0.94	0.95	0.95	0.96	0.96	0.96	0.97	0.98	0.95	0.95	1	1	1	0.99	0.99	0.99	0.99
mHET4	0.73	0.72	0.71	0.82	0.82	0.82	0.78	0.78	0.77	0.77	0.76	0.79	0.79	0.87	0.82	0.95	0.95	0.98	0.99	1	1	0.99	1	0.98
mHET5	0.76	0.94	0.91	0.94	0.94	0.94	0.92	0.91	0.95	0.95	0.9	0.98	0.98	0.96	0.99	1	0.99	1	0.99	1	0.99	0.97	0.99	0.96
mHET6	0.52	0.97	0.99	1	1	1	0.99	1	1	1	1	1	1	1	0.99	0.99	1	1	1	1	1	1	1	1
mHET7	0.48	0.94	0.97	0.97	0.97	0.97	0.98	0.98	0.98	0.98	0.99	0.99	0.99	0.99	0.99	0.99	1	1	1	1	1	1	1	1
mHET8	0.46	0.92	0.95	0.96	0.96	0.96	0.95	0.95	0.97	0.97	0.96	0.99	0.99	0.99	0.99	0.99	1	1	1	1	1	1	1	0.98
mHET9	0.55	0.87	0.89	0.89	0.89	0.89	0.93	0.94	0.96	0.97	0.92	0.93	0.94	0.93	0.99	0.99	0.97	1	0.99	1	1	1	1	1
mHET10	0.48	0.49	0.51	0.49	0.5	0.51	0.56	0.57	0.59	0.57	0.74	0.68	0.66	0.75	0.99	0.98	0.9	1	0.99	1	1	1	1	1
mZNF1	0.74	0.75	0.76	0.9	0.89	0.89	0.93	0.92	0.92	0.91	0.96	0.96	0.96	0.95	0.95	0.95	0.95	0.94	1	1	1	1	1	1
mZNF2	0.85	0.86	0.87	0.89	0.88	0.88	0.93	0.92	0.92	0.91	0.99	0.99	0.99	0.99	0.99	0.99	0.99	1	1	1	1	1	1	1
mReprPC1	0.67	0.91	0.95	0.86	0.94	0.85	0.88	0.9	0.87	0.91	0.96	0.94	0.97	0.98	0.98	0.99	0.99	0.99	0.99	1	0.99	0.99	0.99	0.97
mReprPC2	0.62	0.67	0.66	0.76	0.83	0.89	0.89	0.7	0.96	0.91	0.96	0.99	0.99	0.99	1	0.99	0.99	1	1	1	0.99	1	0.99	0.99
mReprPC3	0.78	0.78	0.77	0.73	0.76	0.79	0.76	0.76	0.77	0.79	0.75	0.88	0.85	0.75	0.89	0.74	0.76	0.76	0.89	1	0.9	0.83	0.79	0.85
mReprPC4	0.7	0.74	0.74	0.74	0.74	0.73	0.73	0.75	0.74	0.74	0.74	0.74	0.74	0.74	0.74	0.74	1	1	1	1	0.74	0.76	0.74	1
mReprPC5	0.32	0.78	0.8	0.88	0.8	0.89	0.94	0.87	0.95	0.91	0.95	1	0.99	1	1	1	1	1	1	1	1	1	1	0.99
mReprPC6	0.35	0.93	0.91	0.98	0.96	0.99	0.98	1	0.97	0.99	0.98	1	1	1	1	1	1	1	1	1	1	1	1	0.99
mReprPC_openC1	0.69	0.81	0.84	0.84	0.87	0.81	0.85	0.81	0.83	0.84	0.81	0.86	0.85	0.85	0.92	0.84	0.97	0.97	1	1	0.99	0.99	1	0.99
mReprPC_openC2	0.73	0.85	0.88	0.88	0.83	0.88	0.84	0.88	0.84	0.97	0.94	0.89	0.93	0.94	0.91	0.95	0.89	0.89	0.9	1	0.95	0.92	0.95	0.91
mReprPC_openC3	0.7	0.92	0.9	0.89	0.89	0.92	0.92	0.93	0.9	0.91	0.94	0.95	0.92	0.96	0.97	0.96	0.99	0.99	0.99	1	0.97	0.98	0.98	0.98
mReprPC_openC4	0.48	0.94	0.95	0.95	0.95	0.96	0.98	0.98	0.97	1	0.99	0.99	0.99	1	1	1	1	1	1	1	0.99	1	0.99	0.98
mOpenC1	0.75	0.74	0.74	0.97	0.98	0.96	0.96	0.95	0.97	0.94	0.97	0.98	0.97	0.99	0.99	0.99	0.98	0.98	0.99	1	0.99	0.98	1	1
mOpenC2	0.75	0.77	0.62	0.96	0.9	0.95	0.96	0.95	0.97	0.99	0.98	0.94	0.98	1	1	0.99	0.99	0.99	1	1	0.99	1	0.99	1
mOpenC3	0.76	0.76	0.78	0.84	0.81	0.79	0.84	0.8	0.83	0.85	0.85	0.79	0.93	0.91	0.91	0.91	0.91	0.91	0.96	1	1	1	1	0.99
mOpenC4	0.5	0.54	0.55	0.62	0.71	0.67	0.79	0.77	0.88	0.83	0.89	0.9	0.84	0.94	0.94	0.93	0.95	0.95	0.88	1	1	1	0.84	0.85
mOpenC5	0.93	0.87	0.9	0.88	0.87	0.88	0.87	0.85	0.93	0.96	0.96	0.88	0.88	0.86	0.87	0.99	0.99	0.99	1	1	0.99	0.99	0.98	0.96
mOpenC6	0.72	0.7	0.74	0.97	0.98	0.99	0.98	0.98	0.98	0.99	0.99	0.99	0.99	0.99	1	1	1	1	1	1	1	1	1	1
mOpenC7	0.69	0.64	0.67	0.96	0.97	0.96	0.97	0.97	0.97	0.97	0.96	0.95	0.96	0.97	1	1	1	1	1	1	1	1	1	1
mOpenC8	0.53	0.54	0.53	0.69	0.69	0.69	0.68	0.69	0.68	0.68	0.68	0.75	0.73	0.71	0.99	1	1	1	1	1	1	1	1	1
mOpenC9	0.34	0.41	0.43	0.46	0.53	0.67	0.7	0.67	0.73	0.75	0.83	0.83	0.86	0.91	0.99	0.97	0.99	1	1	1	0.99	1	0.99	0.9
mEnhA1	0.86	0.86	0.85	0.81	0.82	0.83	0.83	0.95	0.93	0.97	0.85	0.88	0.92	1	1	1	1	0.99	0.99	1	1	0.99	1	0.99
mEnhA2	0.9	0.91	0.92	0.91	0.93	0.93	0.9	0.93	0.97	0.99	0.96	0.99	0.98	0.98	0.99	0.97	0.99	0.96	1	1	0.99	0.98	0.98	0.98
mEnhA3	0.86	0.91	0.94	0.97	0.98	0.99	0.97	0.95	0.98	0.96	0.95	0.96	0.98	0.96	1	0.97	1	1	1	1	0.97	0.98	0.95	0.98
mEnhA4	0.85	0.83	0.91	0.96	0.97	0.96	0.98	0.97	0.95	0.98	0.98	0.99	1	0.99	0.99	0.98	1	0.98	0.98	1	0.99	0.98	0.99	0.97
mEnhA5	0.81	0.87	0.89	0.89	0.89	0.87	0.85	0.86	0.96	0.95	0.95	0.93	0.96	0.99	0.95	0.94	0.92	0.93	0.93	1	0.97	0.96	0.95	0.98
mEnhA6	0.69	0.66	0.72	0.84	0.91	0.83	0.86	0.87	0.85	0.84	0.87	0.85	0.87	0.86	0.84	0.86	0.89	0.88	1	1	1	1	0.95	0.9
mEnhA7	0.83	0.96	0.95	0.92	0.93	0.91	0.89	0.91	0.91	0.93	0.93	0.96	0.93	0.95	0.94	0.98	0.93	0.93	1	1	0.99	0.99	0.96	0.99
mEnhA8	0.61	0.86	0.87	0.88	0.89	0.93	0.91	0.95	0.95	0.94	0.96	0.93	0.96	0.95	0.94	0.94	0.91	0.96	1	1	1	0.96	0.93	0.95
mEnhA9	0.57	0.86	0.9	0.94	0.95	0.96	0.98	0.98	0.97	0.98	0.98	0.98	0.99	0.98	0.98	1	0.99	1	1	1	1	0.99	0.98	0.99
mEnhA10	0.65	0.88	0.95	0.98	0.97	0.99	0.99	0.99	0.98	0.97	0.96	0.98	0.98	1	0.99	0.99	0.99	1	1	1	0.99	0.99	0.98	0.99
mEnhA11	0.71	0.85	0.85	0.85	0.83	0.91	0.9	0.89	0.85	0.85	0.9	0.89	0.93	0.87	0.99	0.92	1	0.98	1	1	0.99	0.96	0.97	0.96
mEnhA12	0.8	0.89	0.89	0.86	0.86	0.96	0.97	0.97	0.98	0.98	1	1	1	0.99	1	1	0.99	0.98	0.93	1	0.95	0.96	0.97	0.95
mEnhA13	0.67	0.88	0.88	0.84	0.82	0.94	0.95	0.94	0.97	0.93	0.94	0.94	0.95	0.95	0.99	1	1	1	1	1	1	1	1	0.99
mEnhA14	0.59	0.67	0.74	0.68	0.76	0.83	0.86	0.86	0.89	0.89	0.89	0.9	0.89	0.89	0.9	0.97	0.96	0.98	0.99	1	1	0.96	1	0.96
mEnhA15	0.64	0.69	0.7	0.78	0.83	0.92	0.9	0.9	0.96	0.96	0.93	0.96	0.92	0.97	0.99	0.99	0.99	1	0.99	1	1	0.99	0.99	0.99
mEnhA16	0.8	0.78	0.79	0.85	0.84	0.92	0.94	0.95	0.98	0.96	0.97	0.99	0.97	0.98	0.99	0.99	0.97	0.99	1	1	0.99	0.98	0.97	0.97
mEnhA17	0.47	0.71	0.69	0.92	0.94	0.94	0.94	0.95	0.95	1	0.99	1	1	1	1	1	1	0.99	0.98	1	0.98	0.98	0.97	0.97
mEnhA18	0.2	0.47	0.46	0.81	0.81	0.96	0.99	0.98	0.98	0.97	0.95	0.97	0.97	0.98	0.97	0.97	0.98	0.97	0.96	1	0.96	0.96	0.96	0.96
mEnhWk1	0.34	0.37	0.29	0.36	0.32	0.39	0.46	0.55	0.99	1	1	1	0.99	1	0.99	0.99	1	1	0.99	1	1	1	1	1
mEnhWk2	0.73	0.72	0.72	0.84	0.87	0.83	0.82	0.81	0.85	0.83	0.81	0.84	0.91	0.95	0.86	0.86	0.85	0.96	0.86	1	0.95	0.93	0.93	0.92
mEnhWk3	0.71	0.82	0.89	0.8	0.83	0.82	0.8	0.93	0.9	0.93	0.94	0.93	0.94	0.93	0.94	0.91	0.93	0.9	0.87	1	0.96	0.97	0.98	0.95
mEnhWk4	0.55	0.67	0.68	0.6	0.6	0.65	0.63	0.64	0.91	0.87	0.92	0.93	0.93	0.98	0.97	0.97	0.98	0.97	0.97	1	0.94	0.95	0.95	0.92
mEnhWk5	0.37	0.43	0.47	0.43	0.44	0.43	0.51	0.48	0.68	0.63	0.69	0.71	0.73	0.9	0.99	0.99	0.99	0.99	0.99	1	0.93	0.93	0.92	0.97
mEnhWk6	0.36	0.59	0.76	0.77	0.85	0.86	0.86	0.89	0.9	0.94	0.94	0.94	0.92	0.92	0.95	1	1	1	1	1	1	1	1	0.96
mEnhWk7	0.38	0.45	0.67	0.63	0.76	0.8	0.83	0.87	0.84	0.91	0.94	0.89	0.99	0.99	1	1	1	1	1	1	1	0.93	1	0.92
mEnhWk8	0.41	0.68	0.68	0.8	0.83	0.81	0.8	0.82	0.82	0.85	0.88	0.89	0.89	0.92	0.98	0.96	0.98	0.99	1	1	0.99	1	1	0.93
mEnhWk9	0.44	0.67	0.68	0.79	0.86	0.93	0.95	0.95	0.94	0.86	0.87	0.87	0.89	0.89	0.89	0.94	0.9	0.93	0.97	1	0.99	0.99	0.99	1
mEnhWk10	0.43	0.5	0.51	0.58	0.75	0.89	0.76	0.79	0.96	0.97	0.99	1	0.99	1	1	0.99	1	1	1	1	1	1	1	1
mEnhWk11	0.58	0.64	0.69	0.63	0.6	0.89	0.96	0.97	0.97	0.94	0.99	0.99	0.99	0.99	0.96	0.99	1	1	1	1	1	1	0.98	0.99
mEnhWk12	0.61	0.82	0.81	0.88	0.88	0.84	0.84	0.84	0.83	0.87	0.87	0.87	0.87	0.86	0.87	0.86	0.86	0.86	0.88	1	0.99	0.98	0.99	0.98
mEnhWk13	0.31	0.45	0.61	0.65	0.73	0.72	0.71	0.71	0.73	0.8	0.82	0.8	0.8	0.82	0.83	0.86	0.99	0.99	0.98	1	0.97	0.96	0.96	0.96
mTxEnh1	0.84	0.88	0.88	0.86	0.88	0.93	0.88	0.86	0.93	0.99	0.96	0.9	0.9	0.9	0.99	0.98	0.99	0.95	0.94	1	0.99	0.98	0.99	1
mTxEnh2	0.56	0.69	0.72	0.77	0.71	0.71	1	0.98	0.97	0.93	0.97	0.99	0.99	1	0.99	0.99	0.99	0.99	0.99	1	1	1	0.99	0.92
mTxEnh3	0.34	0.49	0.67	0.68	0.6	0.62	0.86	0.93	0.93	0.97	0.95	0.92	0.87	0.86	0.88	0.89	0.99	1	0.99	1	0.99	0.99	0.99	0.99
mTxEnh4	0.93	0.92	0.91	0.91	0.91	0.91	0.91	0.99	0.93	0.99	0.99	0.99	0.99	0.99	0.99	1	1	1	1	1	1	1	0.96	0.96
mTxEnh5	0.73	0.72	0.94	0.93	0.93	0.93	0.94	0.96	0.96	0.97	0.95	0.98	0.98	0.98	0.97	0.97	0.97	0.97	0.96	1	0.96	0.93	0.97	0.99
mTxEnh6	0.61	0.7	0.76	0.75	0.77	0.78	0.78	0.81	0.81	0.82	0.9	0.86	0.85	0.85	0.91	0.89	0.88	0.87	1	1	1	1	1	1
mTx1	0.79	0.92	0.92	0.92	0.96	0.96	0.96	0.92	0.93	0.95	0.96	0.95	0.97	0.97	0.97	0.97	0.96	0.96	1	1	0.99	0.99	0.99	0.99
mTx2	0.96	0.97	0.97	0.98	1	1	1	0.99	0.99	0.99	0.99	0.99	0.99	0.99	0.99	0.99	0.99	0.99	1	1	1	1	1	1
mTx3	0.98	0.98	0.99	0.99	0.99	0.99	0.99	1	0.99	0.99	1	1	1	1	1	1	1	1	1	1	1	1	1	1
mTx4	0.97	0.99	0.99	0.99	1	1	1	1	1	1	1	1	1	1	1	1	1	1	1	1	1	1	1	1
mTx5	0.99	0.98	0.97	0.96	0.96	0.96	0.96	0.96	0.98	0.97	0.97	0.99	1	1	1	1	1	1	1	1	1	1	1	1
mTx6	0.9	0.95	0.95	0.94	0.92	0.92	0.92	0.97	0.93	0.97	0.97	0.96	0.96	0.96	0.96	1	1	1	1	1	1	1	1	1
mTx7	0.78	0.83	0.89	0.88	0.95	0.95	0.95	0.93	0.94	0.94	0.93	0.91	0.91	0.91	0.95	0.91	0.9	0.9	0.93	1	1	0.94	0.99	0.99
mTx8	0.66	0.67	0.66	0.67	0.67	0.67	0.67	0.71	0.69	0.87	0.84	0.93	0.96	0.97	0.95	0.91	0.91	0.93	0.99	1	0.99	0.99	1	0.99
mTxEx1	0.94	0.95	0.96	0.97	0.97	0.98	0.97	0.96	0.99	1	1	1	1	1	1	1	1	1	1	1	0.99	0.99	1	1
mTxEx2	0.72	0.73	0.93	0.92	0.94	0.93	0.93	0.96	0.99	1	0.99	0.99	0.99	1	0.99	1	1	1	1	1	0.96	0.95	1	1
mTxEx3	0.54	0.53	0.65	0.63	0.82	0.82	0.81	0.81	0.8	0.8	0.79	0.79	0.84	0.84	0.79	0.85	0.84	1	0.84	1	0.8	0.79	0.96	0.96
mTxWk1	0.46	0.64	0.62	0.62	0.79	0.75	0.74	0.96	0.96	0.95	0.97	0.95	0.83	0.83	0.83	0.84	0.95	0.95	0.99	1	0.99	0.99	1	1
mTxWk2	0.87	0.9	0.9	0.9	0.87	0.89	0.88	0.91	0.91	0.89	0.94	0.89	0.88	0.88	0.87	0.87	0.87	0.87	0.88	1	0.9	0.88	0.91	0.88
mBivProm1	0.54	0.75	0.9	0.9	0.9	0.89	0.91	0.85	0.97	1	0.99	0.98	0.96	0.99	0.99	0.99	0.98	0.98	0.99	1	0.92	0.95	0.93	0.99
mBivProm2	0.73	0.68	0.86	0.86	0.86	0.98	0.97	0.97	0.99	0.98	0.98	0.97	0.97	1	0.97	1	1	1	1	1	0.98	0.98	0.99	0.98
mBivProm3	0.39	0.54	0.76	0.76	0.92	0.79	0.91	0.94	0.93	0.94	0.95	0.94	0.96	0.95	0.97	0.98	1	0.99	1	1	0.98	0.99	1	1
mPromF1	0.87	0.84	0.93	0.93	0.93	0.9	0.93	0.93	0.91	0.92	0.92	0.93	0.92	0.88	0.97	0.98	1	1	1	1	0.99	0.99	1	1
mPromF2	0.96	0.97	0.95	0.95	0.93	0.98	0.99	0.99	0.98	0.99	0.99	0.99	0.99	1	0.99	1	1	1	1	1	1	1	0.99	0.98
mPromF3	0.91	0.86	0.9	0.89	0.9	0.92	0.91	0.92	0.98	0.98	0.9	0.89	0.96	0.97	0.99	1	0.99	1	0.99	1	0.99	0.98	0.97	0.95
mPromF4	0.78	0.73	0.86	0.85	0.81	0.8	0.8	0.78	0.83	0.84	0.78	0.77	0.79	0.83	0.86	0.87	0.97	0.96	0.94	1	0.99	0.97	0.95	0.97
mPromF5	0.79	0.78	0.86	0.86	0.95	0.95	0.95	0.99	1	1	0.99	0.99	1	1	1	1	1	1	1	1	0.96	0.97	0.97	1
mPromF6	0.45	0.48	0.64	0.63	0.91	0.91	0.9	0.98	0.98	0.98	0.98	0.97	0.99	1	0.97	1	1	1	1	1	0.98	0.99	0.95	0.93
mPromF7	0.43	0.39	0.58	0.57	0.6	0.61	0.59	0.68	0.84	0.85	0.85	0.83	0.95	0.89	0.85	0.87	0.93	0.96	0.92	1	0.97	1	0.98	0.99
mTSS1	0.94	0.95	0.91	0.91	0.94	0.93	0.9	0.94	0.95	0.95	0.94	0.93	0.94	1	0.93	1	1	1	1	1	1	0.99	1	0.99
mTSS2	0.85	0.9	0.98	0.98	0.99	1	1	1	1	1	1	1	1	1	1	1	1	1	1	1	1	1	1	1
mTSS3	0.87	0.89	0.89	0.89	0.89	0.88	0.88	0.89	0.88	0.87	1	0.99	1	1	1	1	1	1	1	1	1	1	1	1
column min	0.2	0.37	0.29	0.36	0.32	0.39	0.46	0.48	0.59	0.57	0.68	0.68	0.66	0.71	0.74	0.74	0.76	0.76	0.84	1	0.74	0.76	0.74	0.85
column max	0.99	0.99	0.99	1	1	1	1	1	1	1	1	1	1	1	1	1	1	1	1	1	1	1	1	1

Supplementary Figure 10: Analysis for the number of states. (A) The negative log-likelihood (neg2_log_llh), Akaike Information Criterion (AIC), Bayesian information criterion (BIC) measures (the lower, the better) for models with different numbers of states. We trained models with number of states ranging from 5 to 120, in increments of 5. For each model, the reported measures are calculated on a random set of 300 genomic regions, each of length 1Mbp. These

measures show how well each model can fit the input data, and AIC and BIC also penalizes model complexity. As the number of states increase, neg2_log_llh, AIC and BIC decrease but with diminishing magnitude. **(B)** Correlations between the states within the 100-state model with states in alternative models. For each state in the 100-state model (rows), we calculated the *maximum* correlations between the state (in 100-state model) with states in other models (columns). The heatmap color corresponds to the correlations (1: blue, 0: white). The 95-state model can capture all states in the 100-state model with a minimum maximum correlation of 0.84, and such correlation becomes 0.76 with the 90-state model, as shown in the next to last row.

References

Gorkin,D.U. *et al.* (2020) An atlas of dynamic chromatin landscapes in mouse fetal development. *Nature*, **583**, 744–751.

Kwon,S.B. and Ernst,J. (2021) Learning a genome-wide score of human–mouse conservation at the functional genomics level. *Nat. Commun.*, **12**, 1–14.

Siepel,A. *et al.* (2005) Evolutionarily conserved elements in vertebrate, insect, worm, and yeast genomes. *Genome Res.*, **15**, 1034–1050.

Smit,A.F.A. *et al.* (2015) RepeatMasker Open-4.0. 2013–2015.

Vu,H. and Ernst,J. (2022) Universal annotation of the human genome through integration of over a thousand epigenomic datasets. *Genome Biol.*, **23**, 1–37.
