# Supplementary material for: Universal chromatin state annotation of the mouse genome
Source: Genome Biol. 2023 Jun 27;24:153. doi: 10.1186/s13059-023-02994-x (PMC10294404; doi:10.1186/s13059-023-02994-x)
Supplement: Supplementary file 6 — Additional file 6. Review history. [file 13059_2023_2994_MOESM6_ESM.docx]

Review History

**First round of review**

**Reviewer 1**

**Were you able to assess all statistics in the manuscript, including the appropriateness of statistical tests used?**

There are no statistics in the manuscript.

**Comments to author:**

The authors utilize 901 datasets representative of 26 cell type groups within the mouse model to generate a full-stack chromatin state annotation. Overall, the paper is a valuable contribution with only a few concerns to be addressed.

MAJOR concerns:
1. It is unclear if a 100-state model is justified. In their previous publication for human cells, they used a 100 state model for over a 1000 datasets from more than 100 cell types. In this mouse paper they had 901 datasets from only 26 cell types.
2. The methods used to map the mouse to human chromatin states are not included in the manuscript. This is concerning because only 22/100 states have 50-fold enrichment with at least one human state. The authors should address the limited overlap; are the non-overlapping states unique to mouse or is the mouse dataset and state model missing specific chromatin states found in humans.
Minor Issues:
1. There is no need for the "m" in all the figure and text to represent mouse.
2. Figure 1A lacks the label for the heat map values. The figure legend states the heatmap is for the probability within the state of observing a binary present call. Denoting this on the figure will make it clearer to readers.
3. In Figure 2B, the header "Assoc. LECIF range" looks faded and should be made clearer.

**Reviewer 2**

**Were you able to assess all statistics in the manuscript, including the appropriateness of statistical tests used?**

No, I do not feel adequately qualified to assess the statistics.

**Comments to author:**

In manuscript GBIO-D-22-01813, Vu and Ernst report a full-stack chromatin state annotation of the mouse genome based on the analysis of 901 datasets assaying 14 chromatin marks in 26 different cell or tissue types. The concept and underlying method of full-stack annotation were reported previously by the same authors along with a full-stack annotation of the human genome (Vu and Ernst, Genome Biology 2022). Full-stack annotation complements the current per-cell-type annotation and offers several advantages. For instance, it provides a unified view of all data and can differentiate between constitutive and cell-type-specific activities. Good annotation is essential for the correct interpretation of genome function and full-stack annotation is a welcome and highly innovative addition that deserves to be applied to data from model organisms including from mice in addition to human data.

As the method has been previously published and described in great detail, I have no issues with any technical aspect and focused my comments on general aspects relating to the usefulness of full-stack annotation as a resource.

1. While full-stack annotation extends the repertoire of features that can now be annotated, it is also likely to miss some features. It would therefore be interesting to discuss which features are gained and missed and why? For instance, the full-stack annotation reports three states for enhancers (active enhancers (mEnhA), weak enhancers (mEnhWk), and transcribed enhancers (mTxEnh) but none for e.g. latent enhancers.

2. The ENCODE blacklist describes a comprehensive set of regions in the mouse and other genomes that have anomalous signals independent of the source or experiment. The current recommendation is to remove such regions when analysing functional genomics data. As blacklisted regions were included here and compared with the full-stack annotation, it would be interesting to know if the full-stack annotation has helped to resolve any blacklisted regions?

3. One of the aims of full-stack annotation is to produce a single universal genome annotation which could be an ideal entry point for researchers. While the full-stack annotation is publicly available from Github, I wonder if there are any plans to make it available in a format that allows visual exploration by non-experts, e.g. in a genome browser?

**Authors Response**

**Point-by-point responses to the reviewers’ comments:**

Reviewer #1:

The authors utilize 901 datasets representative of 26 cell type groups within the mouse model to generate a full-stack chromatin state annotation. Overall, the paper is a valuable contribution with only a few concerns to be addressed.

*Response: We thank the reviewer for their summary and positive comments about our manuscript. We also thank the reviewer for their constructive comments, for which we present our point-by-point response to below.*

MAJOR concerns:

1.	It is unclear if a 100-state model is justified. In their previous publication for human cells, they used a 100 state model for over a 1000 datasets from more than 100 cell types. In this mouse paper they had 901 datasets from only 26 cell types.

*Response: We thank the reviewer for this comment. We previously decided to focus on a 100-state model using the 901 datasets in mouse mostly to keep the number of states consistent between the two models, which we felt would facilitate comparisons between the two models. We note the number of datasets used between the models is not too different (1032 vs. 901). Many of the cell types in human model are closely related and form a smaller number of tissue groups (e.g. 19 used in many analyses (Roadmap Epigenomics Consortium et al., 2015)). It is thus not obvious the human input data covers a drastically larger space of biological diversity than the mouse input data.*

*In our revised manuscript, we have conducted further analyses to evaluate the effects of the number of states on the model learning.*

*First, we trained analogous models to the 100-state model presented in the manuscript, such that the number of states ranged from 5 to 120 and are a multiple of 5 (5-, 10-, 15, ..., 120-state models). We then calculated the negative log-likelihood (the lower, the better), Akaike information criterion (AIC) and the Bayesian information criterion (BIC) for all these models, evaluated using input data of 300 genomic regions of length 1-Mbp. The AIC and BIC measures reflect how well the model fit the input data, while also penalizing the model complexity (i.e. number of parameters). As the number of states increase, the negative log-likelihood, AIC and BIC measures all decrease, but with diminishing improvements with increasing numbers of states (see new* ***Supp. Fig. 10*** *shown below).*

*Second, we compared how the emission parameters of non-100-state models correlate with the 100-state model. Here, we used the ChromHMM CompareModels function to evaluate, for each of the 100 states in our presented model, the maximum correlation with one state in each of the alternative models (with different numbers of states). Here, the correlation between two states are calculated across the emission probabilities for the 901 experiments. The analysis shows that a model with 95 states captures the states in the 100-state model with a minimum maximum correlation of 0.84, and this value decreases to 0.76 with the 90-state model.*

*Focusing on 100 states, as with the human model (Vu and Ernst, 2022) allows us to balance the expressivity of a larger model while still having a manageable number of states for interpretation and downstream analyses. In addition, it facilitates comparison between the two models.*

*We have edited the Methods subsection ‘****Training full-stack modeling and generating genome-wide state annotations****’ and added Supplementary Figure 10 to report the analyses related to the choice of number of states, as presented below.*

***Training full-stack modeling and generating genome-wide state annotations***

*We learned the mouse full-stack chromatin state model for the 901 datasets using the LearnModel command of ChromHMM (v.1.23). We applied the same set of flags as in learning the human full-stack model (-splitrows -holdcolumnorder -pseudo -many -p 6 -n 300 -d -1 -lowmem -gzip), described in Vu and Ernst, 2022 (Vu and Ernst, 2022). We trained models with numbers of states ranging from 5 to 120, in increments of 5 states. We then calculated the negative log-likelihood, Akaike information criterion (AIC) and Bayesian information criterion (BIC) measures on a random set of 300 genomic regions, each of length 1Mbp, for all models 5 (****Additional File 1: Supp. Fig. 10****). These measures show how well each model fit with the observed data, while also putting penalty on the model complexity (more states, more model parameters and hence more complex models). We observed that as the number of states increase, the negative log-likehood, AIC and BIC measures (the lower the better) all decreased, but at diminishing magnitude (****Additional File 1: Supp. Fig. 10****). We decided to focus on analyzing a model with 100 states since it is similar to the number of states in the human model, to facilitate comparisons between the two models (Vu and Ernst, 2022). Additionally, using the ChromHMM CompareModels function, we also calculated the maximum correlation between each state in the 100-state model presented here, with a state from each of the alternative models (with different numbers of states) (****Additional File 1: Supp. Fig. 10****). The correlation was calculated across emission probabilities for the 901 experiments. The reported correlations indicate how well each state in the chosen 100-state model can be captured by one state in each of the alternative models. We observed that the minimum correlations between the 100-state model with any other alternative models range from 0.20 (5-state model) to 0.85 (120-state model).*

***Supplementary Figure 10:*** *Analysis for the number of states.* ***(A)*** *The negative log-likelihood (neg2_log_llh), Akaike Information Criterion (AIC), Bayesian information criterion (BIC) measures (the lower, the better) for models with different numbers of states. We trained models with number of states ranging from 5 to 120, in increments of 5. For each model, the reported measures are calculated on a random set of 300 genomic regions, each of length 1Mbp. These measures show how well each model can fit the input data, and AIC and*

*BIC also penalizes model*

*complexity. As the number of states increase, neg2_log_llh, AIC and BIC decrease but with diminishing magnitude.* ***(B)*** *Correlations between the states within the 100-state model with states in alternative models. For each state in the 100-state model (rows), we calculated the maximum correlations between the state (in 100-state model) with states in other models (columns). The heatmap color corresponds to the correlations (1: blue, 0: white). The 95-state model can capture all states in the 100-state model with a minimum maximum correlation of 0.84, and such correlation becomes 0.76 with the 90-state model, as shown in the bottom two rows.*

2. The methods used to map the mouse to human chromatin states are not included in the manuscript. This is concerning because only 22/100 states have 50-fold enrichment with at least one human state. The authors should address the limited overlap; are the non-overlapping states unique to mouse or is the mouse dataset and state model missing specific chromatin states found in humans.

*Response: We thank the reviewer for this comment. We apologize for unintentionally omitting the methods for mapping between the mouse and human chromatin states, which we have now included in the revised version and also include below.*

*We note that a 50-fold enrichment is potentially overly stringent. For instance, if either a corresponding state in human or mouse covers 2% of the genome, it would be impossible for the pair to show a 50-fold enrichment overlap. At a lower 10-fold enrichment, 64 states from the mouse model overlap with a human state. In addition, we also now report that 65 human unique states were maximally enriched for at least one mouse state. Conversely, 59 unique mouse states were maximally enriched for at least one human state. Furthermore, for 38 of the states, there is a 1-1 correspondence between the maximally enriched human state and the maximally enriched mouse state. The majority of these 38 states were mapped to a human state with the same regulatory function category (TSS, flanking promoters, transcriptional activities states, etc.).*

*Some of the discrepancies between the sets of states learned from the human and mouse models can be due to different inputs to the different models. For example, the human model state group ‘Acet’ (8 states) show higher signals of various histone acetylation marks in a small number of biosamples that were not present in the 901 input datasets to the mouse model. The mouse model introduced a new group of states (mReprPC_openC), compared to the human model, that correspond to both open chromatin and repressive activity signals. Additionally, the mouse model contains states corresponding to open chromatin regions with varying degree of cell-type-specificity (states mDNase 1-5,8-9,* ***Additional File 3****). In contrast, the human full-stack model includes only one state (DNase1) of constitutive open chromatin and CTCF insulator enrichments.*

*Finally, we note that the reported fold-enrichment is based on mapping human annotations to mouse through genome sequence information (using liftOver). It is possible states can have similar meanings in both species but not at corresponding positions defined by sequence information. Consistent with this possibility, we observed states from the mouse mHET and human HET group both associate with H3K9me3 and repeat elements despite a limited enrichment overlap in this analysis. We have clarified these points in the revised manuscript.*

*We have now added a subsection within the Methods section titled ‘****Overlap enrichments between mouse and human full-stack states****’ to describe the detailed procedure to map the overlap enrichments between the human and mouse states, shown below:*

*‘****Overlap enrichments between mouse and human full-stack states***

*We first lifted-over the human full-stack annotation from hg19 to mm10 by lifting over each genomic bin of size 200-bp independently using the liftOver tool with minMatch parameter set to 0.1. Then, we used ChromHMM OverlapEnrichment function to calculate the overlap fold enrichment of each mouse state for each human state (liftedOver to mm10).*

*For each pair of human-mouse states, we determined whether the pair of states correspond to a one-to-one mapping by checking whether the human state was most enriched with the mouse state compared to other 99 human states and vice versa for the mouse state.*

*We modified* ***Supplementary Figure 9*** *to add one column showing the one-to-one mappings between mouse and human states.*

***Supplementary Figure 9: Mouse full-stack states’ relationship with LECIF scores, human full-stack states and phastCons elements.*** *LECIF scores were developed to measure the level of evidence of human-mouse conservation at functional/epigenomic levels, with higher score (maximum of 1 and minimum of 0) implies higher evidence of conservation (Kwon and Ernst, 2021). PhastCons elements correspond to genomic regions showing strong 60-way multi-species sequence alignment conservation (Siepel et al., 2005). Human full-stack states were learned from >1,000 Chip-seq/DNase-seq datasets in human, and provide annotation of the human genome that is shared across cell/tissue types (Vu and Ernst, 2022).* ***(A)*** *The heatmap shows mouse full-stack states (rows)’ average LECIF scores, enrichments with phastCons elements and the maximum enrichments with human full-stack states. The first column shows the mouse full-stack states. Coloring of the next 3 columns is column specific, as specified in legend. The last two column shows the human state with the highest enrichment with each of the mouse state, and a star (*) next to a mouse state that is a one-to-one mapping with a human state. Here, one-to-one mapping of states means that the mouse state is most enriched with the human state compared to other 99 mouse states, and vice versa with the human state (****Methods****).* ***(B)*** *Upset plot showing the number of states that are among the top 20 states with either (1) highest average LECIF score, or (2) hig`hest enrichments with PhastCons elements or (3) highest maximal enrichments with human full-stack states. Within each category, the column below the upset plot lists states that are in the top 20 most associated (as measured by average LECIF scores or fold enrichments) with the combination enrichment contexts.*

*We also modified* ***Supplementary Figure 8*** *to add the annotation of mouse full-stack states that were maximally enriched with each human full-stack state. An excel accompanying this figure is provided in* ***Additional File 4****.*

***Supplementary Figure 8: Enrichments of mouse full-stack states with human full-stack states (Vu and Ernst, 2022).*** *The first three columns show mouse full-stack states, the maximum fold enrichment with a human full-stack state (across all human states) and the corresponding human state, respectively. The following columns show the overlap enrichments*

*with of each mouse state (rows) with each human state (columns). Across all pairs of states, the smallest enrichment values are colored blue and enrichment values >= 100 are colored red. The last row shows the mouse states that were maximally enriched with each human state, compared to other 99 mouse states. Thirty-eight out of 100 mouse full-stack states were one-to-one mapped to a human state, with the fold-enrichment ranging from 3.21 (mQuies2 and human Quies1 states) to 378.33 (mTxEx3 and human TxEx4 states). We note that 65 unique human states were maximally enriched with at least one mouse state and conversely, 59 unique mouse states were the maximally state for at least one human state. This figure is also provided in* ***Additional File 4.***

*We also modified the* ***Results*** *section to better clarify this point. All the modifications are shown below:*

*‘In addition, we analyzed how the mouse full-stack states correspond to those of an analogous previously defined full-stack model in human (Vu and Ernst, 2022). We evaluated the enrichments of each mouse full-stack state with each human full-stack state after mapping the human annotations to mouse based on DNA sequence information (****Methods****). Sixty-four and 22 states showed >10-fold and >50-fold enrichment, respectively, with at least one human state (****Additional File 1: Fig. S8-9, Additional File 4****) (Vu and Ernst, 2022), and these states’ biological implications highlight strong correspondence of states from the human and mouse models. For example, mouse state mTxEx3 showed 378.8-fold enrichment for human state TxEx4 – the largest enrichment across any pair of states— (****Additional File 1: Fig. S9, Additional File 4****). These two states showed the highest average gene expression across multiple mouse and human cell types, respectively (Vu and Ernst, 2022) (****Fig. 2C****). All 13 mouse states in the promoter groups (mPromF, mBivProm, mTSS states) showed strong enrichments with human full-stack states that are also promoter-associated, with 12 of these mouse states showing >90-fold enrichment (****Additional File 1: Fig. S9, Additional File 3****). Mouse states mOpenC6-7 showed the strongest association with the human DNase state, and all these states are associated with constitutive open chromatin and CTCF elements (****Fig. 2F, Additional File 3****) (Vu and Ernst, 2022). However, there exist differences between states from the two organisms’ models. For example, state group mReprPC_OpenC (showing DNase-seq and/or ATAC-seq and repressive mark H3K27me3 signals) was only observed in the mouse model, while the group Acet (showing chromatin acetylation signals) was only present in human model, which was as expected as most of these acetylation marks were not in the mouse model input. Seven states in the mOpenC group (all except mDNase6-7) showed cell-type-specific signals of open chromatin, a pattern not observed in the human model (****Additional File 1: Fig. S8-9, Additional File 4****). Other states such as those in the mouse mHET and human HET groups shared an association with H3K9me3 and enrichments for repetitive elements, but did not show strong enrichments in this analysis. This was expected because the mapping of human and mouse states was based on lifting-over positions with corresponding DNA sequences between the two species.’*

Minor Issues:

1. There is no need for the "m" in all the figure and text to represent mouse.

*Response: We thank the reviewer for this suggestion. While it is true the ‘m’ prefix is redundant in differentiating states within the mouse model, we find the ‘m’ prefix helpful in drawing a clear distinction among states learned between the human and mouse models. This is important since there can be some differences in interpretation between a state from the mouse model and a state*

*from the human model with the same label. Therefore, to avoid confusion for cases where both models could be considered, we prefer to include ‘m’ prefix to the mouse model’s states.*

2. Figure 1A lacks the label for the heat map values. The figure legend states the heatmap is for the probability within the state of observing a binary present call. Denoting this on the figure will make it clearer to readers.

*Response: We thank the reviewer for this comment. We have included labels for the heatmap values, as shown in the revised Fig. 1A below.*

3. In Figure 2B, the header "Assoc. LECIF range" looks faded and should be made clearer.

*Response: We thank the reviewer for pointing this out. We have fixed this issue in the updated Fig. 2 as shown below.*

Reviewer #2:

In manuscript GBIO-D-22-01813, Vu and Ernst report a full-stack chromatin state annotation of the mouse genome based on the analysis of 901 datasets assaying 14 chromatin marks in 26 different cell or tissue types. The concept and underlying method of full-stack annotation were reported previously by the same authors along with a full-stack annotation of the human genome (Vu and Ernst, Genome Biology 2022). Full-stack annotation complements the current per-cell-type annotation and offers several advantages. For instance, it provides a unified view of all data and can differentiate between constitutive and cell-type-specific activities. Good annotation is essential for the correct interpretation of genome function and full-stack annotation is a welcome and highly innovative addition that deserves to be applied to data from model organisms including from mice in addition to human data.

As the method has been previously published and described in great detail, I have no issues with any technical aspect and focused my comments on general aspects relating to the usefulness of full-stack annotation as a resource.

*Response: We thank the reviewer for the summary and their positive comments about the manuscript. We also thank for their constructive comments to which we present our point-by-point response below.*

1. While full-stack annotation extends the repertoire of features that can now be annotated, it is also likely to miss some features. It would therefore be interesting to discuss which features are gained and missed and why? For instance, the full-stack annotation reports three states for enhancers (active enhancers (mEnhA), weak enhancers (mEnhWk), and transcribed enhancers (mTxEnh) but none for e.g. latent enhancers.

*Response: We thank the reviewer for this question. The full-stack annotation offers complementary advantages, compared to existing per-cell-type annotations, such as learning states that correspond to either constitutive or cell-type-specific epigenetic contexts or simplifying genome annotation across different cell types, etc. In cases where researchers are interested in a particular cell type, then per-cell-type annotation is more suitable for the investigation, so that researchers will not miss any epigenetic features in the cell type of interest. We have previously discussed the advantages and ideal use cases for the full-stack annotation in (Vu and Ernst, 2022).*

*In this manuscript we did analyze the probabilities of overlap between each of the full-stack states with each of the states in per-cell-type annotations, for 66 different mouse reference epigenomes (of different tissue types and developmental stages)* ***(Supplementary Figures 6-7****). This analysis investigates how each full-stack state can capture the chromatin states present in cell types that had been extensively profiled through ChIP-seq and ATAC-seq experiments (Gorkin et al., 2020).*

*If the reviewer is defining latent as regions that lack enhancer-related histone marks but gain them upon stimulation (e.g. (Ostuni et al., 2013)), the extent to which the stacked modeling approach can capture latent enhancers will depend upon the specific model inputs. When researchers can obtain data of chromatin mark signals in cell types undergoing different treatments, the framework of stack-modelling of ChromHMM can be applied to learn states that may correspond to epigenetic states that are switched on/off in different cell conditions.*

*We have modified the Conclusion section to clarify on these points, as below:*

*‘We introduced the mouse full-stack annotation to provide a universal chromatin state map using >900 epigenomic datasets from 26 cell type groups. The mouse full-stack model and its characterization is analogous to the previous human full-stack model (Vu and Ernst, 2022) (****Data Availability, Additional File 2****). As discussed previously, the full-stack model has a number of advantages, such as being able to differentiate constitutive from cell type-specific annotations and simplifying the overall genome annotation across cell types, in that there is a single genome annotation per position (Vu and Ernst, 2022). However, this does come at a trade-off of a more complex set of model parameters and potential loss of modeling power when one is interested in a specific cell type. The full-stack annotation is not meant to replace existing per-cell-type annotations, but rather to complement them and the most appropriate annotation will likely depend on the application (Vu and Ernst, 2022). Here, we applied the stacked modelling approach to data from different cells/tissues, and the same modeling approach could be applied to data measured within the same cell type but under different treatments to potentially uncover latent enhancers (Ostuni et al., 2013). We expect the full-stack model to serve as an additional resource for work that leverages mouse as a model organism to gain insight into human biology.’*

2. The ENCODE blacklist describes a comprehensive set of regions in the mouse and other genomes that have anomalous signals independent of the source or experiment. The current recommendation is to remove such regions when analysing functional genomics data. As blacklisted regions were included here and compared with the full-stack annotation, it would be interesting to know if the full-stack annotation has helped to resolve any blacklisted regions?

*Response: We thank the reviewer for this question. Two states, GapArtf1 and GapArtf3, stand out as being highly enriched with blacklisted regions, 5.4 and 5.0-fold, respectively (****Fig. 2A****). In total, there are 413,502,000 bp annotated as either state GapArtf1 or GapArtf3, while there are 238,977,200 bp annotated as being in a blacklisted region based on ENCODE. There are 193,765,600 bp in the intersection of (1) blacklisted and (2) GapArtf1 or GapArtf3, constituting 81.1% of blacklisted regions. The Jaccard index between blacklisted region and GapArtf1/3 regions is 0.42 (193,765,600 bp of intersection and 458,713,600 bp of union). Blacklisted regions that fall into GapArt1 or GapArtf3 have additional support to their classification. Regions that are unique to either set would need a more careful manual curation for resolving their classification.*

*Based on this analysis, we have modified corresponding portions in the Results and Methods sections accordingly.*

*In the Results section:*

*‘States mGapArtf1 and mGapArtf3 jointly overlapped with 81.1% of the blacklisted regions from ENCODE (****Fig. 2A, Methods****).’*

*In the Methods section:*

- *Blacklisted regions were downloaded from ENCODE project portal in mm10 from (Amemiya et al., 2019). Two states stand out in their enrichments with blacklisted regions, GapArtf1 and GapArt3. In total, 413,502,000 bp are annotated as either state GapArtf1 or GapArtf3, 238,977,200 bp are annotated as the blacklisted region. The Jaccard index*

*between blacklisted region and GapArtf1/3 regions is 0.42 (193,765,600 bp of intersection, constituting 81.08% of all blacklisted region, and 458,713,600 bp of union).*

3. One of the aims of full-stack annotation is to produce a single universal genome annotation which could be an ideal entry point for researchers. While the full-stack annotation is publicly available from Github, I wonder if there are any plans to make it available in a format that allows visual exploration by non-experts, e.g. in a genome browser?

*Response: We thank the reviewer for the question. We previously provided a version of the annotations that could be loaded as a custom track into genome browsers. To make it even easier for non-expert users to view the annotations in a browser, we have now made a custom track hub for viewing both the human and mouse full-stack annotation on UCSC genome browser. We provide a link to this custom track hub along with associated detailed step-by-step instructions from the project’s Github page (*[*https://github.com/ernstlab/mouse_fullStack_annotations).*](https://github.com/ernstlab/mouse_fullStack_annotations).)

*We have modified the ‘Data Availability’ section accordingly.*
*‘****Data Availability***

*The mouse full-stack chromatin state annotation, along with detailed instructions for viewing the annotation on the UCSC Genome Browser through the provided trackhub link, are available at* [*https://github.com/ernstlab/mouse_fullStack_annotations*](https://github.com/ernstlab/mouse_fullStack_annotations) *in mm10. The code to analyze the full-stack states are available at* [*https://github.com/ernstlab/mouse_fullStack_annotations*](https://github.com/ernstlab/mouse_fullStack_annotations)*. All download links for input for the mouse full-stack model are available in* ***Additional File 2****.’*

***References***

*Amemiya,H.M. et al. (2019) The ENCODE blacklist: identification of problematic regions of the genome. Sci. Rep.,* ***9****, 1–5.*

*Gorkin,D.U. et al. (2020) An atlas of dynamic chromatin landscapes in mouse fetal development. Nature,* ***583****, 744–751.*

*Kwon,S.B. and Ernst,J. (2021) Learning a genome-wide score of human–mouse conservation at the functional genomics level. Nat. Commun.,* ***12****, 1–14.*

*Ostuni,R. et al. (2013) Latent enhancers activated by stimulation in differentiated cells. Cell,* ***152****, 157–171.*

*Roadmap Epigenomics Consortium et al. (2015) Integrative analysis of 111 reference human epigenomes. Nature,* ***518****, 317–330.*

*Siepel,A. et al. (2005) Evolutionarily conserved elements in vertebrate, insect, worm, and yeast genomes. Genome Res.,* ***15****, 1034–1050.*

*Vu,H. and Ernst,J. (2022) Universal annotation of the human genome through integration of over a thousand epigenomic datasets. Genome Biol.,* ***23****, 1–37.*

**Second round of review**

**Reviewer 1**

The authors have addressed my concerns.

**Reviewer 2**

The authors have satisfactorily addressed all the points raised in my original review.
